# α-Synuclein: An All-Inclusive Trip Around its Structure, Influencing Factors and Applied Techniques

**DOI:** 10.3389/fchem.2021.666585

**Published:** 2021-07-07

**Authors:** Nicolò Bisi, Lucia Feni, Kaliroi Peqini, Helena Pérez-Peña, Sandrine Ongeri, Stefano Pieraccini, Sara Pellegrino

**Affiliations:** ^1^ BioCIS, CNRS, Université Paris Saclay, Châtenay-Malabry Cedex, France; ^2^ DISFARM-Dipartimento di Scienze Farmaceutiche, Sezione Chimica Generale e Organica “A. Marchesini”, Università degli Studi di Milano, Milan, Italy; ^3^ Dipartimento di Chimica, Università degli Studi di Milano, Milan, Italy

**Keywords:** intrinsically disordered protein, synucleinopathy, secondary and tertiary structure, protein interaction, *in silico* studies

## Abstract

Alpha-synuclein (αSyn) is a highly expressed and conserved protein, typically found in the presynaptic terminals of neurons. The misfolding and aggregation of αSyn into amyloid fibrils is a pathogenic hallmark of several neurodegenerative diseases called synucleinopathies, such as Parkinson’s disease. Since αSyn is an Intrinsically Disordered Protein, the characterization of its structure remains very challenging. Moreover, the mechanisms by which the structural conversion of monomeric αSyn into oligomers and finally into fibrils takes place is still far to be completely understood. Over the years, various studies have provided insights into the possible pathways that αSyn could follow to misfold and acquire oligomeric and fibrillar forms. In addition, it has been observed that αSyn structure can be influenced by different parameters, such as mutations in its sequence, the biological environment (e.g., lipids, endogenous small molecules and proteins), the interaction with exogenous compounds (e.g., drugs, diet components, heavy metals). Herein, we review the structural features of αSyn (wild-type and disease-mutated) that have been elucidated up to present by both experimental and computational techniques in different environmental and biological conditions. We believe that this gathering of current knowledge will further facilitate studies on αSyn, helping the planning of future experiments on the interactions of this protein with targeting molecules especially taking into consideration the environmental conditions.

## Introduction

**FIGURE 1:**
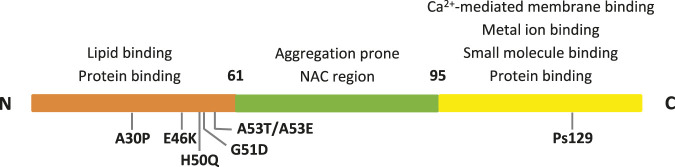
The three αSyn regions are represented in different colors. Their properties, along with the main PD mutations and interacting sites are indicated.

Alpha-synuclein (αSyn) is a relatively small protein formed by 140 residues, which is highly expressed and conserved. It is typically found in the presynaptic terminals of neurons. Its primary sequence can be divided into three regions, as shown in Figure 1 (Fusco et al., 2014; Mori et al., 2020; Uversky and Eliezer, 2009) that are characterized by different physico-chemical properties due to their distinct aminoacidic composition. First, the *N*-terminal segment, (residues 1–60), shows numerous amphipathic 11-mer repetitions, and contains the consensus sequence KTKEGV. This is the αSyn region where most of the familial mutations are located. Then, the non-amyloid-β-component (NAC) central region (residues 61–95) is highly amyloidogenic giving the protein the ability to generate β-sheets. Finally, the *C*-terminal segment (residues 96–140) is rich in anionic residues and prevents αSyn aggregation by electrostatic repulsion.

In its native state, monomeric αSyn is unfolded, and thus is commonly considered as an intrinsically disordered protein (IDP). Yet, there is still a large controversy regarding αSyn secondary and tertiary structural tendencies and the data from literature are often conflicting. Changes in the environment conditions, mutations, interactions with endogenous and/or exogenous molecules can indeed induce αSyn to fold in different structures. αSyn misfolding and its subsequent aggregation into amyloid fibrils is a pathogenic hallmark of different synucleinopathies, such as Parkinson’s disease (PD). As a consequence, the comprehension of αSyn structural and functional features is fundamental to progress in the study and finding of treatments for αSyn-related diseases.

Here, we provide a review on *in silico* and experimental data regarding the structural features of αSyn both in the WT form and in biologically relevant mutants. Moreover, we focus on different factors influencing αSyn structure, such as the biological environment, the interaction with lipids, with endogenous small molecules and proteins, as well as with exogenous compounds (e.g., drugs, diet components, heavy metals). We also discuss the different methods used to highlight αSyn structure in each case and the relation between the obtained results and the employed technique.

## Monomeric Wild Type (WT) αSyn Structural Features

In 1996, Weinreb et al. observed that Wild-Type (WT) αSyn exists in solution as a dynamic ensemble of conformations lacking a single equilibrium structure and, therefore, classified it as an IDP (Weinreb et al., 1996). Many studies have advanced our knowledge in this field by applying experimental (for a recent review on NMR investigations see Kim et al., 2020) and computational (e.g., MD, Monte Carlo simulations) techniques (Jónsson et al., 2012), or a combination of both approaches (Brodie et al., 2019). However, due to αSyn structural heterogeneity that depends on many different biological and physico-chemical factors (Stephens et al., 2019), caution is needed when interpreting these results. To date, the general consensus is that monomeric WT αSyn is almost unstructured in solution (Fauvet et al., 2012). Anyway, variations of WT αSyn structural propensity can be detected. In order to rationalize the vast amount of literature data, we try to categorize them according to two different levels: global and local (Table 1 and below).

**TABLE 1:** Reported experimental and computational data on monomeric wt αSyn.

Structural features		Technique		References
Global	Local	Experimental	Computational	
Compact, globular structure Electrostatic interactions (120–140 and 30–100 residues)	–	Technique: PRE^1^	Technique: MD^2^	Dedmon et al. (2005a)
		Spin label MTSL		
		Cysteine mutations at Q24, S42, Q62, S87 and N103		
Brief long-range intramolecular electrostatic interactions	–	Technique: NMR^3^	–	Bertoncini et al. (2005)
		100uM WT αSyn in buffer (25mM Tris.Cl pH = 7.4/0.1M NaCl)		
Extended tendency	N-terminal: Helical elements	Technique: NMR^3^	Technique: restrained MD^2^	Allison et al., 2009
Brief long-range intramolecular electrostatic interactions			Solvent: implicit	
Compact structure at both neutral and low pH	–	Technique: PRE^1^	–	Wu and Baum, (2010)
C- to N-terminal interchain interactions		Various pH, concentrations, solvents		
Compact structure (low pH)	–	Technique: Single-Molecule FRET^4^	Technique: constrained excluded volume MC^5^	Nath et al. (2012)
		50 pM solution of double-labeled WT αSyn	Technique: All-atom MD^2^	
			Solvent: explicit	
High-energy phase: Extended random coil Low-energy phase: Extended all-β	High-energy phase:	–	Technique: MC^5^	Jónsson et al. (2012)
	N-terminal: Helical elements		Solvent: Implicit	
	Low-energy phase:			
	N-terminal + NAC + C-terminal (residues 30–100): β-strands			
	C-terminal: β-structures + random coil			
–	N-terminal + NAC + C-terminal (residues 1–100): Helical elements	–	Technique: REMD^6^	Coskuner et al., (2013)
			Solvents: implicit	
			Technique: MD^2^	
			Solvents: explicit	
Compact structure	N-terminal: Helical elements + β-hairpin spanning residues 38–53	–	Technique: coarse-grained MD^2^	Yu et al. (2015)
Electrostatic interactions (118–130 and 38–53 residues)	β-strands: β1 (38–44) and β2 (47–53)		Solvent: explicit	
α+β	NAC: Helical elements			
	C-terminal: Helical elements + β-structures			
Compact, globular structure	N-terminal: Helical elements	Technique: HS-AFM^7^	Technique: REX^8^/DMD^9^	Zhang et al. (2018)
Tail-like protrusions	NAC: Helical elements	50nM WT αSyn in PBS	Solvent: Lazaridis-Karplus implicit	
All-α	C-terminal: Helical elements			
Compact, globular structure	N-terminal: Helical elements spanning residues 25–55	Techniques:	Technique: REX^8^/DMD^9^	Brodie et al., 2019
Brief long-range intramolecular	NAC: β-structures	LD-CL^10^, CD^11^,HDX^12^,	Solvent: Lazaridis-Karplus implicit	
electrostatic interactions α+β	C-terminal: β-structures + random coil	LC-MS^13^/MS Analysis		
Compact, globular structure	N-terminal: Helical elements	–	Technique: MD^2^	Bhattacharya et al. (2019)
Tail-like protrusions	NAC: Helical elements		Solvents: explicit	
All-α	C-terminal: Helical elements + random coil			
–	N-terminal: Helical elements spanning residues 10–30 and a weak helix centered around residue 50	Technique: NMR^3^	–	Kim et al. (2020) (review)
	NAC: tendency to form β-structures helix centered around residue 90			

1 Paramagnetic Relaxation Enhancement.

2 Molecular Dynamics simulations.

3 Nuclear magnetic resonance.

4 Fluorescence resonance energy transfer.

5 Monte Carlo simulations.

6 Replica Exchange Molecular Dynamics simulations.

7 High-Speed Atomic Force Microscopy.

8 All-atom Replica Exchange.

9 Discrete Molecular Dynamics.

10 Long-distance crosslinking.

11 Circular dichroism.

12 Hydrogen-deuterium exchange.

13 Liquid chromatography–mass spectrometry.

### Global-Level

#### Tertiary Structure Propensity

**FIGURE 2:**
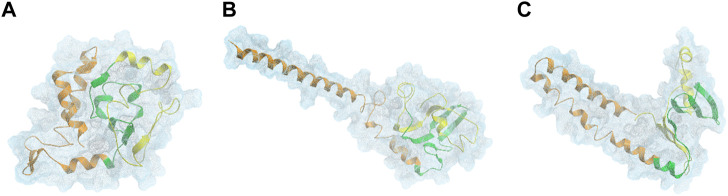
DMD centroids of the most frequent monomeric WT αSyn lowest energy clusters. Clusters representing a **(A)** ∼76%, **(B)** 15%, and **(C)** ∼4% of the overall population. αSyn N-terminal region (residues 1–60) is colored in orange, the NAC-region (residues 61–95) is colored in green and the C-terminal region (residues 96–140) is colored in yellow (Zhang et al., 2018).

**FIGURE 3:**
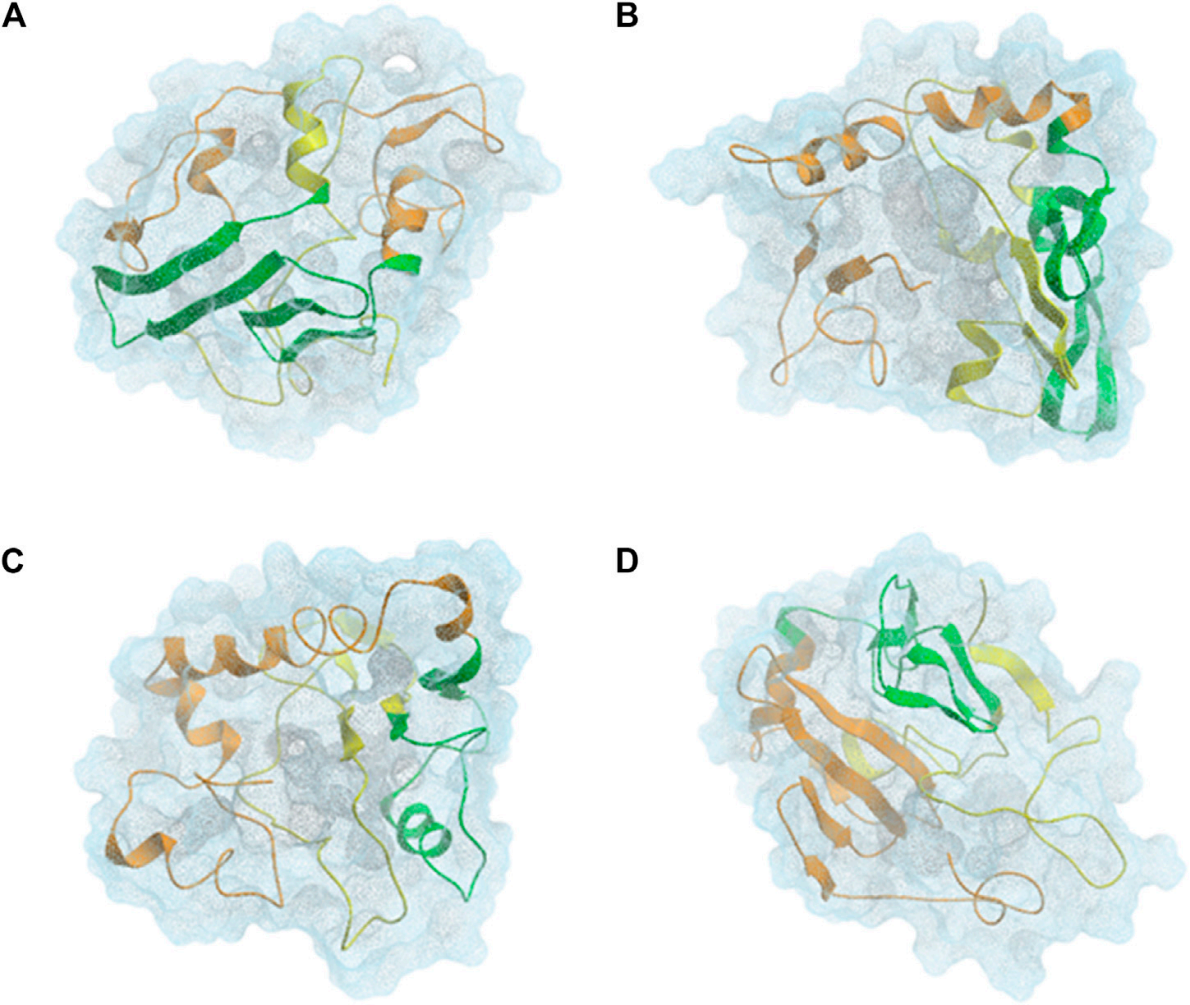
CL-DMD WT monomeric αSyn models. Centroids from the most relevant conformational clusters containing **(A)** 37%, **(B)** 28%, **(C)** 20%, and **(D)** 8% of the overall population. αSyn *N*-terminal region (residues 1–60) is colored in orange, the NAC-region (residues 61–95) is colored in green and the *C*-terminal region (residues 96–140) is colored in yellow (Brodie et al., 2019).

αSyn is able to interconvert between multiple states of the dynamic ensemble of conformations (Weinreb et al., 1996). Nonetheless, at the global-level, different research groups have reached different conclusions as to whether the conformational ensemble in solution is on average: (1) likely to be compact and acquire a globular-like structure driven mainly by long-range intra-molecular electrostatic interactions, as illustrated in Figures 2 and 3 or (2) prone to exist as an extended random coil.

At physiological pH, WT αSyn has a very uneven distribution of physico-chemical properties along its sequence. The *N*-terminal region is amphipathic, the NAC region hydrophobic, and the *C*-terminal region highly negatively charged (Ilie and Caflisch, 2019). Flickering structural tendencies can be observed when viewing the hydrophobic effect as the major driving force for protein folding (Kauzmann, 1954). Based on this assumption, the contacts between the hydrophobic residues and the polar solvent are minimized. In turn, the regions formed by hydrophilic residues, such as the polar part of the *N*-terminal region and the *C*-terminal region, are expected to be more exposed to the cellular solvent and transiently interact with each other (Dułak et al., 2020). Experimental and computational techniques have suggested the presence of brief long-range intramolecular electrostatic interactions within αSyn structure (Dedmon et al., 2005b; Bertoncini et al., 2005; Allison et al., 2009; Fakhree et al., 2018; Brodie et al., 2019).

Dedmon et al. and Yu et al. lack of agreement on the exact residues that form the αSyn intra-electrostatic contacts, nonetheless, both agree on the existence of such interactions between the residues present in the *C*-terminal domain and those located in the central part of the protein. Moreover, Bertoncini et al. (2005) reported that perturbation of these long-range naturally occurring interactions could lead to the exposure of the NAC region (residues 61–95) toward the cellular environment, potentially promoting αSyn oligomerization. Furthermore, *in vivo* and *in vitro* experiments have shown that the truncation of the monomeric WT αSyn *C*-terminal region can induce the formation of amyloid aggregates (Iyer et al., 2017; Vasili et al., 2019). Hence, several studies hypothesized that these detected intra-molecular contacts reduce the accessibility of the central part of the protein, preventing it from establishing inter-molecular interactions and inhibiting monomeric WT αSyn oligomerization and aggregation. As a consequence, some authors have rationalized the possibility of αSyn *C*-terminal region demonstrating a protective role against the formation of amyloid fibrils (Dedmon et al., 2005b; Bertoncini et al., 2005; Yu et al., 2015).

The temperamental nature of these aggregation-resistant globular conformations can be affected by changes in the environment; for example, changes in pH alters the distribution of charges throughout αSyn, which can lead to the loss of these transient intra-molecular electrostatic interactions. The *C*-terminal domain, (residues 96–140), presents a high content of acidic residues at physiological pH which are thought to play a major role inhibiting αSyn aggregation (Dedmon et al., 2005b; Bertoncini et al., 2005; Bhattacharya et al., 2019). Studies suggest that, this self-inhibition against fibrillation conformation can be lost when changing the pH from neutral to acidic (Plotegher et al., 2014).

Contrarily, Nath et al. observed that αSyn acquires a more compact conformation at low pH (Nath et al., 2012). However, this should be taken with caution as these are predictions and not conclusive observation. Moreover, some research groups point out that the interactions between the *C*-terminal region and the rest of the molecule is rather small and, therefore, the contacts established within the native structure provide limited protection against solvent exposure for the NAC region (Jónsson et al., 2012).

Further criticism suggests that monomeric WT αSyn acquires more extended or tail-like global conformations, which aligns with the fact that it is unstructured in solution. Zhang et al. reported the structural dynamics of αSyn in aqueous solution, demonstrating its ability to interchange its structure dynamically, mainly between the primary overall globular morphology and both one-tail and two-tail structures. These tails are parts of the protein that protrude from the main globular segment (Figure 2) (Zhang et al., 2018). The tendency of αSyn to adopt a tail-like structure has also been reported by other researchers, based on MD simulations, Small-Angle X-ray Scattering (SAXS) and Electron Microscopy (EM) experiments (Tsigelny et al., 2012; Lorenzen et al., 2014). These transient tail-like structures are often seen in IDPs because they are implicated in diverse biological functions (Uversky, 2013).

Lastly, other studies stated that, in aqueous solvent, monomeric WT αSyn has a weak preference for adopting globular conformations (Weinreb et al., 1996; Ilie and Caflisch, 2019). For instance, Allison et al. observed that, over time, monomeric WT αSyn has a propensity to expand (Allison et al., 2009). Also, others have reported relevant clusters of αSyn monomers detected in their experiments presenting extended conformations (Jónsson et al., 2012).

The controversial results about the globular or extended preferences obtained by various groups may be ascribed to the rapid interconversion between conformers affecting αSyn and the use of different methodologies to carry out their investigations.

#### Overall Secondary Structure Propensity

**FIGURE 4:**
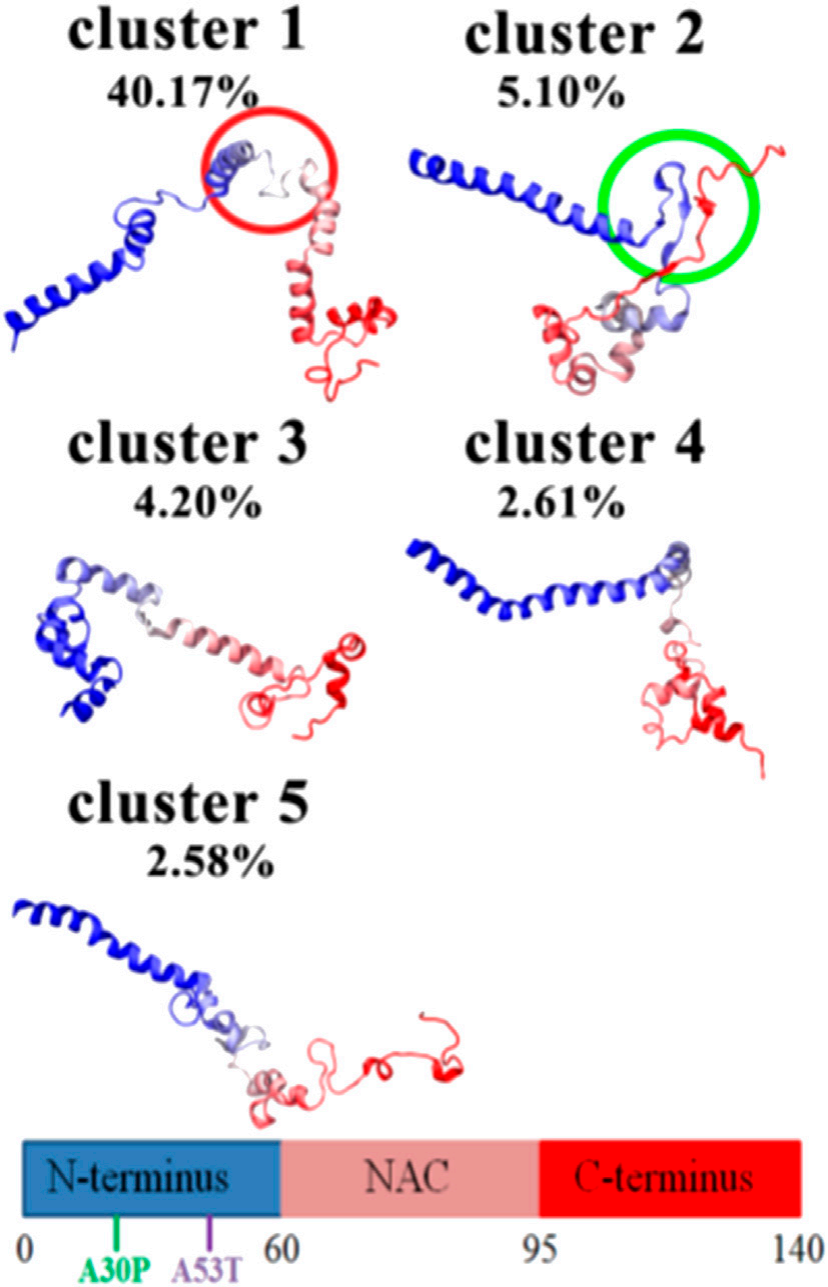
MD WT monomeric aSyn models. Centroid from the most relevant conformational clusters. This figure has been reproduced upon copy right permissions (Yu et al., 2015).

Knowing that monomeric WT αSyn is unstructured in solution, the classification of its transient secondary structural elements can be useful for further investigations. Attempts to determine a structure of native αSyn has been mainly classified it as an all-α protein, whose secondary structure is composed exclusively of α-helices allowing a small number of isolated β-sheets (Figure 2) (Bhattacharya et al., 2019; Cartelli et al., 2016; Meade et al., 2019; Zhang et al., 2018) or as an α+β protein when along the αSyn backbone α-helices and β-strands are intercalated (Figures 3 and 4) (Yu et al., 2015; Brodie et al., 2019).

Conversely, Jónsson et al. predicted that αSyn could adopt an all-β secondary structure in which αSyn was almost entirely composed by β-sheets with some peripherical small α-helices in several of the detected relevant conformers obtained.

They also reported that these results match with experimental data obtained at neutral pH and low temperatures, around 15°C (Jónsson et al., 2012). Zhang et al. (2018) found this conformational extended β-sheet pattern to be unfavorable in the WT αSyn monomeric state.

Some studies argue that folded helical conformers are not anticipated to be pathogenic (Meade et al., 2019) and impede amyloidogenic aggregation (Bhattacharya et al., 2019) whereas the presence of β-sheets drive this process. In fact, the design of novel small molecules or biological therapeutics to stabilize α-helical monomers is a strategy for blocking the neurotoxic pathway switching off β-sheet structure formation (Plotegher et al., 2014; Ciechanover and Kwon, 2015).

### Local-Level

Since we face the problem of the lack of technical resources to irrefutably determine a series of conformations that full-length WT αSyn monomers can adopt in solution, attempts have been made to use available techniques to identify structural trends at the local-level, that is, if it even has a determinable structure. Despite its unstructured nature, αSyn can be analyzed in terms of its transient secondary structures. This allows us to hypothesize the conformational changes αSyn undergoes before the molecular aggregation process is carried out and identify possible target-sites that facilitate the design of drugs to avoid the formation of amyloid fibrils in earlier stages. As a matter of fact, due to the intrinsic dynamic equilibrium of this protein in solution, as mentioned, different tools and techniques for proteins characterization capture different structural trends. However, there is an identifiable trend in which several research groups outline that the *N-*terminus of WT αSyn is prone to fold into a helical conformation, whereas the *C*-terminus contains many fragments found as random coils. There is less agreement as to whether the NAC region folds into β-sheets, which is the key secondary structure that directs protein aggregation, or whether it maintains a helical structure.

#### *N*-Terminal Region

Monomeric WT αSyn N-terminal region can adopt different transient secondary structural features in aqueous solution due to its intrinsically disordered nature. Several studies observed a tendency in the αSyn *N*-terminus to acquire a helical secondary structure (Vilar et al., 2008; Allison et al., 2009; Jónsson et al., 2012; Coskuner and Wise-Scira, 2013; Zhang et al., 2018; Bhattacharya et al., 2019; Brodie et al., 2019; Meade et al., 2019; Kim et al., 2020). This helical pattern has been proposed to be essential for vesicle and membrane binding (Coskuner and Wise-Scira, 2013; Vasili et al., 2019). Hence, this local conformation is prone to be energetically favorable, especially in the presence of factors known to drive this helical structural feature, such as acidic negatively charged membranes (Vasili et al., 2019).

Contrarily, Jónsson et al. predictedan αSyn conformational low-energy phase in solution, in which residues spanning from 30 to 100 contained a high average strand population (Jónsson et al., 2012). Additionally, Yu et al*.* identified in residues 38–53 a high probability of assuming a β-hairpin conformation, formed by antiparallel β-strands β1 (38–44) and β2 (47–53), connected by a turn in region 44–47 (Figure 4) (Yu et al., 2015). This protein region includes some of the residues belonging to two of the five αSyn segments suggested to be involved in the core of αSyn fibrils (37–43, 52–59) (Vilar et al., 2008). Other computational and bioinformatic studies have also reported a higher propensity for regions 38–40 and 50–53 to form β-strand structures (Vilar et al., 2008).

#### NAC Region

There is presently no clear agreement as to whether the NAC region (residues 61–95) adopts a helical or a β-sheet structure or, indeed, whether it acquires a structure at all. As this region is involved in triggering protein aggregation, its structure depends to a great extent on the environmental conditions. This is probably why it contains numerous distinct energetically favorable secondary structures.

A combination of experimental and computational approaches (Brodie et al., 2019) and NMR measurements (Eliezer, 2009) have seen a tendency of the αSyn NAC region to form β-structures (Figure 3) (Kim et al., 2020). This supports the significance of the presence of these transient structures in the native protein, alluding to their resemblance to hairpins that form inter-molecular interactions in amyloid fibrils constituting the core of this mature fibrillar form of αSyn (Tuttle et al., 2016; Guerrero-Ferreira et al., 2018). In contrast, in previous NMR studies, it was not possible to detect free αSyn conformations that would lead to the formation of partially folded aggregation intermediates (Wu and Baum, 2010).

Since the αSyn aggregation process is extremely slow, ensemble solution techniques such as NMR may not succeed in identifying the molecules that are prone to drive this process because they may appear in very small percentages (Plotegher et al., 2014). Nonetheless, Zhang et al. (2018) via HS-AFM documented that the extended β-sheet pattern in the WT αSyn monomeric state is unfavorable.

Other experiments show the sporadic formation of helical structures in the NAC region (Figure 2) (Coskuner and Wise-Scira, 2013; Zhang et al., 2018)

#### *C*-Terminal Region

The *C*-terminus of IDPs has been identified as the most important region since it has numerous functionalities (Uversky, 2013). It follows that this protein area adopts different structures depending on the function that it is required to perform. The results of the investigations that have tried to structurally characterize αSyn in an aqueous medium, either by experimental or computational methods, indicate that the *C*-terminal end tends to present a random coil structure for the most part under physiological conditions (Jónsson et al., 2012; Brodie et al., 2019).

Despite this tendency to present fewer secondary structure elements than the other protein regions, a propensity to form β-structures (Eliezer, 2009; Jónsson et al., 2012; Yu et al., 2015) and helical elements (Lorenzen et al., 2014; Yu et al., 2015; Zhang et al., 2018) has been observed in some studies.

## Effect of PD Mutations on αSyn Structural Features

αSyn is a protein involved in PD, not only as the main component of Lewy bodies, but because of its several mutations observed in PD patients. It is well known that mutations can change the phenotype, having several effects on the structure of a protein. Understanding how PD mutations affect αSyn structure and its functions is thus essential for gaining a profound understanding of the protein itself and for developing more effective pharmacological strategies.

### A53T, A30P, and E46K Mutations

In 1997, Polymeropoulos et al. (1997) identified the A53T mutation in the αSyn gene in an Italian kindred and in three unrelated families of Greek origin with autosomal dominant inheritance for the PD phenotype. A year after, Krüger et al. (1998) reported the A30P mutation in the αSyn gene. A third mutation, namely E46K, was identified in 2004 (Zarranz et al., 2004). During the years, it has been highlighted that mutations could impact both the free state of αSyn and its aggregated form. In this context, studies were performed using different techniques such as NMR spectroscopy, CD, and FTIR.

Initial CD studies on WT αSyn and the first two identified mutations, A30P and A53T, showed that the three proteins lack a preferred conformation in solution (Conway et al., 1998; Narhi et al., 1999; Serpell et al., 2000). However, in 2001, by conducting NMR studies, Bussell and Eliezer reported, that the mutation A30P strongly attenuates the helical propensity of the *N*-terminus. They observed indeed a positive C^α^ secondary shift, indicative of a significant preference for helical secondary structure in the WT 18–31 sequence, which was absent in mutant A30P. Conversely, A53T mutation leaves this region unperturbed, exerting a more modest and local influence on structural propensity (Bussell and Eliezer, 2001). In particular, the A53T mutant exhibited a slightly enhanced local preference for extended, β-sheet-like conformations around the site of the mutation. Other NMR studies on the WT, A30P and A53T, revealed a similar β-sheet-rich core region spanning residues 38–94 in the sequence of the two mutants, whereas the *C*-terminus remained flexible and unfolded in both cases (Heise et al., 2005).

McLean et al. investigated the αSyn long-range interactions by fluorescence resonance energy transfer (FRET). They reported, for both the WT and mutant A53T, a weak interaction between the *N*-terminal and *C*-terminal regions, whereas for mutant A30P they observed a statistical increase in the magnitude of FRET signal, indicating a closer vicinity between the *N*- and *C*- terminal regions (McLean et al., 2000).

In 2007, Fredenburg et al. reported a similar random coil secondary structure for both E46K and WT αSyn when free in solution, as highlighted by CD experiments (Fredenburg et al., 2007). In 2009, Rospigliosi et al. studied the effect of mutation E46K on the long-range interactions by paramagnetic relaxation NMR(PRE) and residual dipolar coupling (RDC) measurements. Surprisingly, no decrease in long-range contacts was detected in the mutant E46K with respect to the WT. Furthermore, an increased interaction between the *C*-terminal tail, the NAC and the *N*-terminal regions was observed. The same experiments on A30P and A53T did not indicate any changes in the long-range structure. In the same work, the authors observed a slight increase in local helix propensity in the area immediately adjacent to the mutation of mutant E46K, by calculating its C^α^ chemical shifts deviations in comparison to the deviations of the random coil ones (Rospigliosi et al., 2009).

**FIGURE 5:**
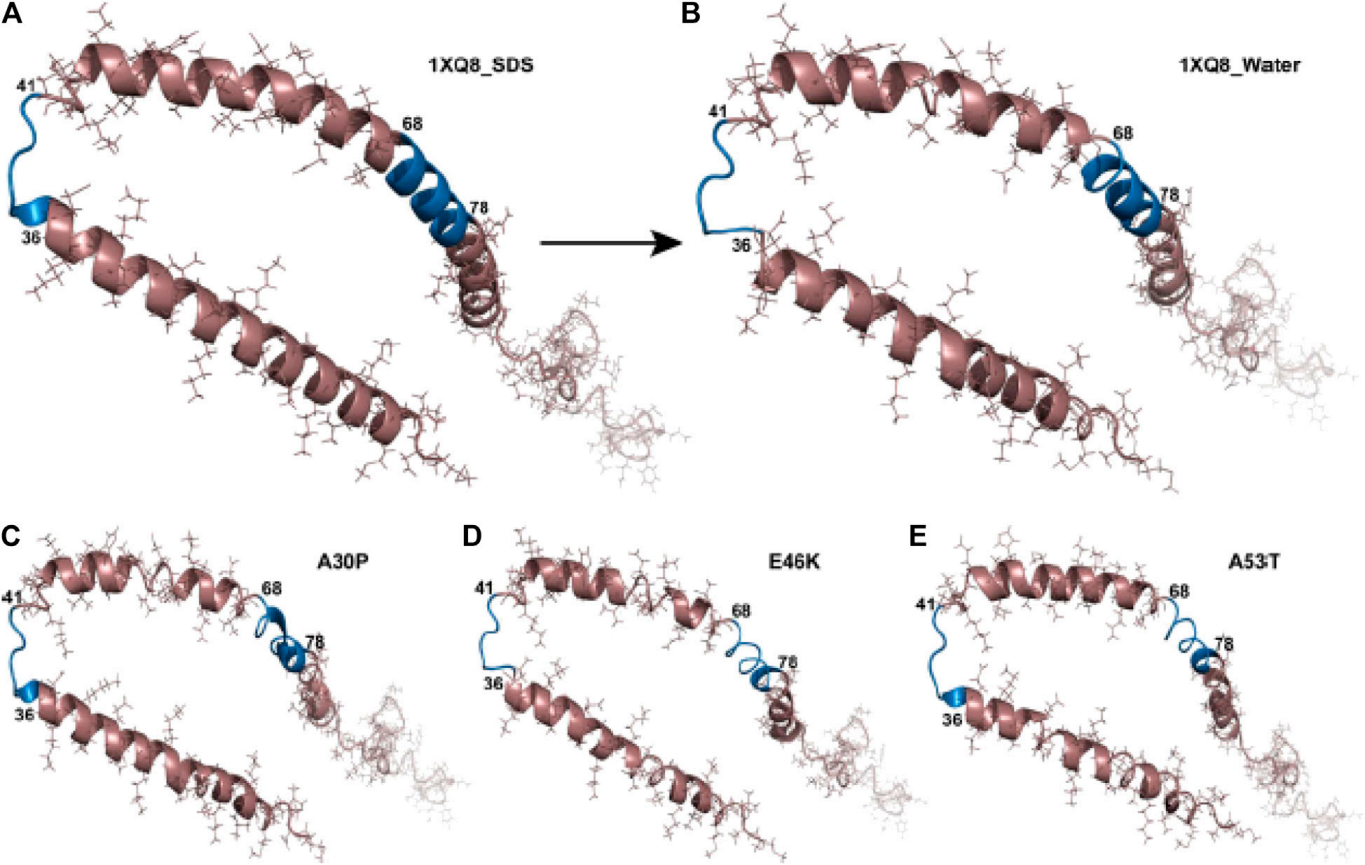
Structural changes in αSyn and the hydrophobic regions. Structure of **(A)** wild type (1XQ8) in SDS solvent, **(B)** wild type (1XQ8) simulated in explicit water conditions, **(C)** A30P mutant in water solvent, **(D)** E46K mutant in water solvent, **(E)** A53T mutant in water solvent. The blue region depicts the 36–41 and 68–78 hydrophobic regions in αSyn showing aggregation propensity (Kumar et al., 2009).

Kumar et al. used Molecular Dynamics (MD) to analyze the mutations A30P, A53T and E46K in water under explicit solvent conditions. These mutants showed variations, more specifically their RMSD scores were 0.529, 0.534, and 0.486 respectively, in their secondary structure compared to WT micelle-bound αSyn (PDB ID 1XQ8) simulated in sodium dodecyl sulfate (SDS) (Figure 5). The secondary structure of A53T recorded in this study was similar to that determined by quenched hydrogen/deuterium exchange NMR spectroscopy which states that five β-strands appear in the amyloid state of αSyn (Vilar et al., 2008; Kumar et al., 2009).

Passing from the last decade to the current one, computational techniques started being more intensively employed to shed light on the structures of the WT and the mutants. In 2011, Balesh et al. (2011) performed classical MD and annealing MD (AMD) simulations and reported similar helical and β-sheet contents for the WT and A53T mutant-type αSyn proteins. At the same time, A53T presented a more compact structure. In 2013, Coskuner and Wise-Scira performed all-atom replica exchange molecular dynamics (REMD) simulations on the full-length monomeric WT and A53T mutant-type αSyn proteins in aqueous solution utilizing implicit and explicit water models. From these results, they observed that the helical content is minimally affected by the mutation A53T except for a few residues in the *N*-terminal and *C*-terminal regions. Additionally, in contrast, to previous computational works (Kumar et al., 2009) they reported an increase in the β-sheet formation close to the mutation site in the *N*-terminal region (Coskuner and Wise-Scira, 2013).

In the same year, a similar MD study was published on mutant A30P by Wise-Scira et al., reporting that the mutation has local as well as long-range effects on the protein structure. More specifically, the helical content of region 18–31 is less prominent in mutant A30P than in the WT protein. The β-sheet structure abundance decreases in the *N*-terminal region upon mutation A30P of the WT αSyn, whereas the NAC and *C*-terminal regions possess larger tendencies for β-sheet structure formation. Long-range intramolecular protein interactions are less abundant upon mutation A30P, especially between the NAC and *C*-terminal regions, leading to a less compact and less stable structure with respect to the WT (Wise-Scira et al., 2013).

### Recently Discovered Mutations

In 2013, a fourth mutation, namely H50Q, was identified (Appel-Cresswell et al., 2013; Kiely et al., 2013). Far-UV CD studies demonstrated that also the H50Q variant is a primarily unfolded protein in aqueous buffers (Chi et al., 2014; Ghosh et al., 2013; Khalaf et al., 2014). Also, Chi et al. (2014), by using heteronuclear single quantum coherence (HSQC) NMR observed that the chemical shifts of most residues between the WT and H50Q were unperturbed, although the *C*-terminal region of H50Q is more flexible than that of the WT. On the contrary, Ghosh et al. noticed chemical shift perturbations between WT αSyn and H50Q, by conducting the same experiments. In fact, they observed quite significant chemical shift perturbations in the mutation area and in the C-terminal region (Ghosh et al., 2013).

In 2014, a fifth mutation, G51D, was discovered (Kiely et al., 2013; Lesage et al., 2013). Fares et al. performed CD experiments where the WT and G51D proteins exhibited the same random coil secondary structure. The ^1^H, ^15^N-HSQC studies confirmed the lack of a preferred conformation for both proteins, while the analysis of the secondary structure propensity *via* C^α^ secondary shifts deviations showed no significant loss or gain of secondary structure compared to the WT. Furthermore, it was observed that the mutation G51D also does not significantly perturb transient long range contacts between *N-*and *C-*termini (Fares et al., 2014).

In the same year, mutation A53E was identified in a Finnish family (Pasanen et al., 2014). Ghosh et al. performed NMR studies with the WT, A53T, and A53E αSyn. Their data showed approximately similar spectra of the WT, A53T, and A53E with relatively narrow dispersions in the proton dimension for all proteins, characteristic for unfolded structures. The chemical shift differences, however, suggest perturbation of chemical shifts for residues surrounding the A53E mutation site, as already observed for the other mutants. Significant chemical shift changes were also observed for the residues at the extreme *C-*terminus of αSyn. In contrast to chemical shift perturbation data, the secondary structural propensity did not show any major alteration due to mutation A53E or A53T (Ghosh et al., 2014).

### Comparative Experiments on all Known Mutated Sequences

**FIGURE 6:**
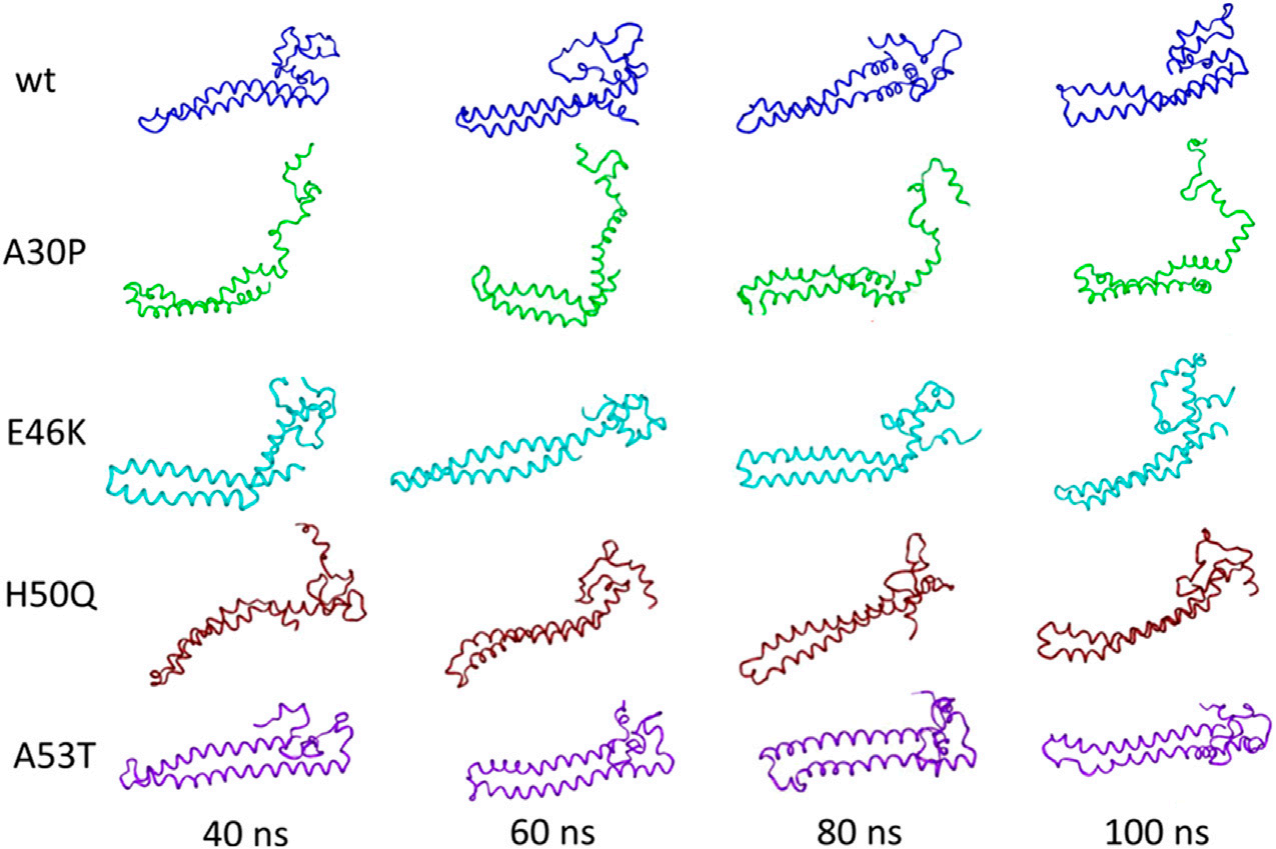
Tertiary structure of the initial NMR conformations of the mutants containing a significant percentage of α-helices changes during MD. Figure modified from Tsigelny et al. (2015).

Recently, Tsigelny et al. generated by MD multiple structural conformations of the WT and all the different mutants, by developing a new combined modeling approach. In the beginning, they simulated WT αSyn and mutant conformers creating a 20-ns interval MD snapshot (Figure 6). From their analysis it can be deduced that the general α-helical content does not change more than 20% in all cases and that these α-helices transform into turns and loops within specific regions for each mutant over the 100 ns of the MD (Tsigelny et al., 2015).

In 2020, Okuwaki et al. examined all the NMR parameters, including the chemical shift and amide-proton exchange of the WT and the mutants. They observed in WT an α-helix structure in the 18–31 fragment, and a β-structure at the C-terminal region 120–140. The β-structure was destabilized by the mutations A30P and A53T. On the other hand, the α–helical structure might be stabilized by these mutations (Okuwaki et al., 2020).

Taken together, these data seem to point out that, among all the observed PD mutations, only A30P affects the overall αSyn structure. In addition, long-range interactions are less abundant. The contact between *N*- and *C*-terminal regions is thus perturbed and it might facilitate the aggregation.

## Effect of the Biological Environment on αSyn Structural Features

**FIGURE 7:**
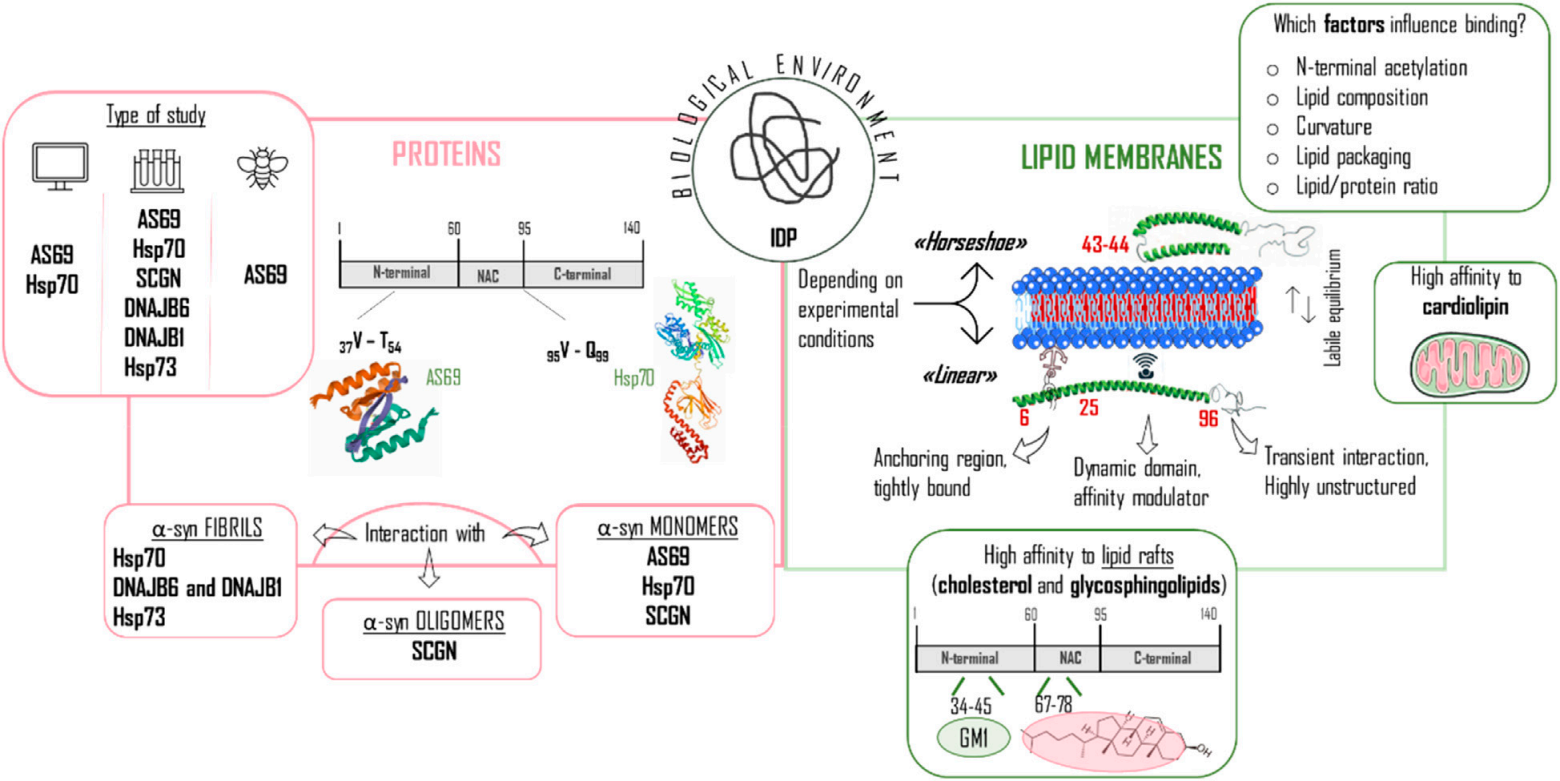
Different biological factors/modulators influencing αSyn structure. The figure is composed of two sections referring to proteins **(left panel)** and lipid membranes **(right panel)**, which have an important role in the conformational change of αSyn from being an intrinsically disordered protein (IDP) to induce a more stable structure. Right panel: special emphasis is placed on the type of study performed, classified in computational, *in vitro* and *in vivo* experiments, and on the particular interaction of the proteins with the distinct αSyn configurations (fibrils, oligomers, monomers). Hsp70 and Hsp73 heat shock protein 70 and 73; SCGN: secretagogin. Left panel: The equilibrium between horseshoe and linear conformation is highlighted and the partition of αSyn in three regions is depicted to show the different behavior throughout the sequence when in contact with lipid membranes. The important interaction with mitochondrial membranes and lipid rafts is also mentioned here. GM1: monosialotetrahexosylganglioside.

From the previous paragraphs, it can be deduced that the structure of the monomeric WT αSyn protein in solution tends to acquire diverse transient and dynamic conformations. αSyn will be likely to adopt specialized conformations upon different conditions (e.g., changes in pH, temperature, ionic strength, closeness to surfaces, etc…) in order to carry out certain biological or pathological functions. Hence, even though the study of WT αSyn conformation alone is useful, a more profitable course of action is to observe the conformational changes induced by different biological and physico-chemical factors triggering structure modifications. In this context, the interaction with endogenous molecules is an important factor to consider. It is commonly accepted that αSyn can bind lipids and phospholipids, as well as several proteins. Here below in Figure 7, the general features are presented, together with several highlights on the interaction with endogenous small molecules.

### Lipids

In living organisms, lipids are mainly used as structural components in cell membranes, as energy stores or as signaling molecules. Various studies have investigated the possibility of αSyn monomer to bind lipids, in particular lipid membranes (plasma and mitochondrial membrane, axonal transport vesicles) (Sung and Eliezer, 2018). In the following paragraphs, we will present an overview of the most recent results, focusing our attention on the protein structure and the innovative strategies and techniques used to obtain these outcomes.

#### Phospholipids

Based on the specific binding properties to lipid layers and the location in synaptic nerve endings, the physiological function of αSyn has been related to circulation and transport of synaptic vesicles (Burré, 2015). Nevertheless, besides its physiological role in the synaptic transmission, the interaction with lipids can also lead to structural changes undergoing aggregation and contributing to amyloidogenesis. Membranes have been reported to both accelerate and inhibit αSyn fibril formation. In fact, the helical fold has been suggested to stabilize the protein and prevent aggregation by hindering the structural transition to β-sheet (Högen et al., 2012) but, at the same time, the helical state has also been proposed as being an intermediate in the aggregation process because it stabilizes intermolecular interactions through hydrophobic contacts (Eliezer et al., 2001). In the latter case, cell membrane surfaces would act as a fibrillation template favoring nucleation and participating in the fibrillation cascade while the NAC region would be essential for the self-polymerization of the protein (Pineda and Burré, 2017; Martial et al., 2019; O’Leary and Lee, 2019). A fact that argues in favor of the role of αSyn/lipid interaction in the etiopathogenesis of synucleinopathies is that all missense mutations responsible for familial PD (e.g., A30P and E46K) are localized in the 11-residue repeat domain; indeed, these mutations alter the lipid binding properties modifying membrane interaction (Bodner et al., 2010; Robotta et al., 2017). Therefore, it is particularly critical to understand how this interaction can regulate the equilibrium between the soluble intrinsically disordered monomer and the structured membrane-bound monomer/oligomer *in vivo*.

The first preliminary hypothesis about αSyn binding to membranes was developed by Davidson et al. (1998), who reported αSyn binding to acidic small unilamellar vesicles (SUVs), underlining the importance of membrane charge and curvature. In particular, an increase in α-helicity from 3 to 80% was measured. Browne and coworkers first tried in 2001, by means of modern multi-dimensional heteronuclear NMR spectroscopy, to characterize the conformational properties of αSyn as a free monomer and when bound to lipid-mimetic SDS detergent micelles and lipid vesicles. A prevalent disposition toward α-helical conformation in the *N*-terminal region was suggested in the free monomer in comparison to the *C*-terminus that, on the contrary, displays a highly unfolded and extended structure. Not surprisingly this tendency could be fulfilled after association to phospholipids, showing an extended α-helical structure stretching among residues 1–100 (Eliezer et al., 2001). Few years later, this idea of an extended conformation was revised by Chandra et al., who asserted that the *N*-terminal region, interacting with SDS, surprisingly configures itself in two helical regions that are interrupted by a short break around residues 43–44, as demonstrated by NMR studies and proteolysis experiments. This interruption has been explained with a more favorable binding of hydrophobic residues to the interior of the membrane or, alternatively, a more advantageous binding to highly curved vesicles (Chandra et al., 2003). By means of following studies based on EPR, Jao et al. could successfully provide important details about αSyn interaction with lipid bilayers, emphasizing the influence of the membrane features on the conformation of the membrane bound αSyn. The authors observed an extended, curved α-helical structure that is significantly different from the antiparallel helices formed in the presence of the detergent SDS (Jao et al., 2008). However, the experimental evidences provided by EPR are also consistent with different binding modes, involving an extensive membrane rearrangement, as suggested by Bodner et al. (Bodner et al., 2009). In this context of contradictory results, Robotta et al. (2011) presented αSyn as a coexistence of the two conformations: extended α-helix and horseshoe, i.e. two antiparallel α-helices, even if with a preference toward the extended form. The authors obtained these results by site-directed spin labeling in combination with pulsed electron paramagnetic resonance on large unilamellar vesicles (LUVs) and they concluded that the two conformations are closely related to the experimental conditions used and that the equilibrium is very labile, which means the molecule is highly flexible. In this way, they could explain why previous studies were in opposition (Robotta et al., 2011).

The interconversion between these two states has been represented as functionally relevant to the protein; in fact, physiologically, αSyn could effectively connect a synaptic vesicle to the plasma membrane by switching from an extended state to a broken-helix conformation (more tightly bound state). For this purpose, in order to characterize the extended helical structure by high-resolution solution-state NMR, a fluorinated alcohol (HFIP) has been employed, able to induce a highly helical state. Indeed, the central region corresponding to the non-helical linker displays a certain instability in the helical structure suggesting the possibility of this transition (Sung and Eliezer, 2018).

Solid state NMR (ssNMR) helped in providing new insights in the structural conformation of the membrane-bound αSyn. In fact, since the *N*-terminus is tightly bound to the lipid bilayer, these residues cannot be identified by solution NMR because they are invisible. The results of these experiments on acidic SUVs indicated that residues 6–25 are tightly bound to the membrane and no differences are detected when 1,2-dioleoyl-sn-glycero-3-[phospho-rac-(1-glycerol)] (DOPG) SUVs are used, even if the calculated affinity between αSyn and these vesicles is higher. We already know that the most dynamic part of the molecule is identified with the *C*-terminal region and INEPT (insensitive nuclei enhanced by polarization transfer) MAS (magic-angle spinning) measurements enabled to also characterize this domain as highly unstructured. In general, three domains could be identified: an anchoring *N*-terminal region (6–25), followed by an intermediate dynamic domain (26–96) and an unstructured *C*-terminal domain that only transiently interact with the membrane surface. The central region is shown to be critical in modulating the affinity for the membrane surface and it is subsequently called membrane “sensor.” Furthermore, MAS measurements indicated that the binding occurs at the surface of the membrane and not in the membrane bilayer (Fusco et al., 2014). Following ssNMR experiments helped also to understand the contribution of *N*-terminal acetylation on αSyn. This post-translational modification leads to a stronger membrane affinity and an increased propensity to adopt α helical structures in the *N*-terminal region. According to Runfola et al., *N*-terminal acetylation seems to regulate the binding affinity of αSyn for synaptic vesicles without altering the structural properties of the bound state (Runfola et al., 2020). Considering all these determinant aspects affecting the binding of αSyn to membranes, one can easily understand how the protein binding is sensitive to the experimental conditions used. In particular, a physiological environment should be used to mimic the naturally occurring features and obtain an ultimate description of the αSyn monomer when bound to membranes. In this framework, another factor to be taken into consideration is the influence of calcium ions on the membrane binding propensity of αSyn. This ion, localized at the presynaptic terminals, is able to bind to the *C*-terminus and favors its binding to lipid membranes, as verified by CEST-NMR experiments, leading to the so-called “double anchor mechanism” emphasizing its role in neurotransmitter release (Lautenschläger et al., 2018).

Summarizing, experimental evidences of association with lipid membranes support the strong dependence of the binding on the lipid composition and surface curvature. In general, αSyn binding to membrane is based on electrostatic interactions between the cationic groups of the basic *N*-terminal region (rich in Lys residues) and the anionic phospholipids, which in fact represent excellent models to mimic synaptic vesicles.

Similar considerations can be done for the αSyn fragment 71–82, included in the NAC region, as observed by Bédard et al., who described its role on the structural and assembly behavior of αSyn. As deduced from CD and IR measurements, in the presence of 1-palmitoyl-2-oleoyl-sn-glycero-3-phosphocholine (POPC) membranes, the fragment is mostly disordered as it is in solution, but when in contact with negatively charged membranes (1-palmitoyl-2-oleoylglycero-3-phosphoglycerol, POPG) the peptide adopts an intermolecular parallel β-sheet configuration (Bédard et al., 2014). In a more recent study, its behavior has been analyzed in the presence of partially anionic membranes to mimic in the best way neuronal membranes and an in-register configuration could be validated by means of IR and ssNMR DQF-DRAWS experiments. The amyloid aggregation driver is the electrostatic interaction as it happened also with the *N*-terminal sequence (Martial et al., 2020).

In addition to the curvature degree, the interaction of αSyn and membranes is also regulated by lipid packaging. In view of this, Stöckl et al*.* proved by confocal microscopy that αSyn preferentially interacts with liquid-disordered giant unilamellar vesicles (GUVs): the binding requires anionic lipids in a liquid disordered state, and this is in good correlation with the synaptic vesicles composition (Stöckl et al., 2008). This is also valid for fragment 71–82 (Martial et al., 2020). A comprehensive model for the interaction of αSyn with lipid bilayers has been proposed by Ouberai et al. based on many converging independent studies and new results generated by the combination of dual polarization interferometry, atomic force microscopy and CD spectroscopy. Connecting to membranes with strong curvature and stressed surfaces (cone-shaped lipids), αSyn monomers are apparently able to close the packing defects. In fact, after binding of αSyn to the phospholipid polar heads and insertion of the hydrophobic residues, lipids are induced to laterally expand provoking membrane remodeling and this process is promoted in the presence of packing defects or imperfections (Ouberai et al., 2013). αSyn ordering effect on the membrane has been also investigated by fluorescence anisotropy and it has been concluded that this is concentration dependent and it occurs in the liquid-crystalline state and not in the gel phase. This means that αSyn is able to stabilize the membrane of synaptic vesicles and thereby can be essential to prevent the premature vesicle fusion to the presynaptic membranes (Pirc and Ulrih, 2015). The higher binding affinity to fluid compared to gel phases has been also investigated by Galvagnion et al. by means of CD and DSC studies, suggesting that the higher exposure of hydrophobic area is essential for the binding. Notably, the authors also asserted that shorter and more soluble lipids greatly improve αSyn aggregation and, consequently, its pathological effect (Galvagnion et al., 2016).

Together with the lipid composition, the lipid to protein ratio is a discriminant factor for amyloidogenesis, being able to switch the equilibrium between physiological and pathological paths, as first described by Galvagnion et al. (2015) and later discussed in a detailed review by Kiechle et al. (2020). When this value is high, due to the low local concentration of αSyn, the aggregation can be suppressed. On the contrary, when this value is low or intermediate, αSyn-bound monomers could lead to nucleation and amyloid formation (Terakawa et al., 2018).

Although αSyn has a mainly cytosolic distribution, its ability to adhere to cell membranes, predisposes it to have other cellular localizations. A certain selectivity of αSyn toward mitochondrial membranes has been observed and this propensity has been related to the abundance of the phospholipid cardiolipin. Nevertheless, cardiolipin is mostly present in the inner membrane of the mitochondria. It has been demonstrated that αSyn enters mitochondria *via* import channels and not *via* direct interaction with the lipids of the outer membrane and afterward it is localized in the inner membrane (Zigoneanu et al., 2012). On the other hand, recent studies demonstrated that cardiolipin translocates to the outer mitochondrial membrane in response to cellular stress and binds αSyn species. In this position, cardiolipin can also pull αSyn monomer away from oligomeric/fibrillar aggregates and facilitate its refolding in α-helix. Cardiolipin exposure is therefore a key signal in PD pathogenesis (Ryan et al., 2018). In agreement to these results Ghio et al. also demonstrated that cardiolipin enhances αSyn lipid membrane binding and also favors the membrane pore-forming activity of αSyn oligomers (Ghio et al., 2019).

#### Lipid Rafts

In general, it seems that αSyn specifically binds to anionic phospholipids, when these are embedded in liquid-disordered domains (Stöckl et al., 2008). Nevertheless, various studies demonstrate that lipid rafts can also have a very important role in αSyn binding. Lipid rafts are specialized areas of the plasma where tightly packed cholesterol and sphingolipids accumulate, surrounded by more fluid phospholipids. In fact, these dynamic microdomains adopt a liquid-ordered state and float in the remaining liquid-disordered plasma membrane (Sezgin et al., 2017). Fortin et al*.* demonstrated by a double fluorescent labeling that αSyn specifically associates with lipid rafts and this interaction can be crucial for its synaptic localization and physiological function (Fortin et al., 2004). Furthermore, many other publications illustrated how lipid rafts are closely connected with neurodegenerative diseases (Sebastião et al., 2013; Canerina-Amaro et al., 2019; Mesa-Herrera et al., 2019; Grassi et al., 2020).

In this framework, the analysis of αSyn interaction with cholesterol and gangliosides is fundamental since both have been considered as critical elements that could synergically favor the insertion of αSyn in lipid rafts and influence its pathological and physiological function (Fantini et al., 2011).

##### Cholesterol

Together with phospholipids, cholesterol plays an important role in regulating permeability and fluidity of the membrane. As expected, it also interacts with αSyn modulating its binding to synaptic-like vesicles, cholesterol being a very important component of these structures (Pfrieger, 2003). In particular, two domains of αSyn were recognized by Fantini et al. to bind cholesterol. Especially, residues 67–78 display a high affinity binding with a tilt angle of 46°, as measured by MD. Notably, they asserted that the tilted peptide could probably insert in the membrane and intercalate with the apolar regions of cholesterol leading to a higher affinity. On the contrary, residues 37–43 probably just associated to the hydroxyl group of cholesterol (Fantini et al., 2011). Other authors showed how cholesterol reduced or completely blocked, depending on the concentration used, αSyn binding to non-anionic membranes but, at the same time, this effect was much lower in the presence of negatively charged membranes (Shvadchak et al., 2011). Recently, surface plasmon resonance (SPR) was employed to measure the binding of αSyn monomers to lipid vesicles and this resulted decreased with the addition of cholesterol molecules to the membrane composition. The effect was detected also in the presence of negatively charged vesicles (Jakubec et al., 2019). A very recent publication from Fusco and coworkers confirmed these results showing by CD experiments that αSyn binding is reduced in the presence of cholesterol. However, the weaker interaction was detected by CEST (chemical exchange saturation transfer) experiments only at the NAC region. The previously described property of αSyn of binding two different membranes at the same time, the “double anchor mechanism”, has been also evaluated by DLS (dynamic light scattering) and the ability of αSyn to interact with two vesicles was promoted with the increasing concentration of cholesterol showing that the NAC region is effectively crucial in this step modulating this important biological property (Man et al., 2020). At the same time, Jakubec et al. questioned whether cholesterol could influence αSyn fibrillation and they observed that the aggregation was effectively promoted by analyzing ThT (thioflavin T) and TPE-TPP (bis(triphenylphosphonium) tetraphenylethene) fluorescence assays. This outcome could be explained taking into consideration that cholesterol could act as a nucleation site (Jakubec et al., 2019).

##### Glycosphingolipids

The interaction of αSyn with glycosphingolipids, in particular gangliosides as GM1, has been reported in many articles, revealing their key role in the physiological and pathological function of this protein (Chiricozzi et al., 2020). In 2006, Martinez et al. concluded from SEC (size-exclusion chromatography) HPLC, CD and TEM (transmission electron microscopy) that αSyn displays a very high binding affinity and specificity toward GM1, in comparison to the other gangliosides (Martinez et al., 2007). Differently from cholesterol, the high-affinity binding site of αSyn to glycosphingolipids includes residues 34–45 (Fantini et al., 2011). This affinity is even intensified when αSyn is *N*-acetylated and at the same time the fibrillation is reduced together with enhancement of the helical folding propensity (Bartels et al., 2014). Based on these outcomes, Schneider et al. reported that GM1 displays neuroprotective effects after *in vivo* administration with a decreased αSyn aggregation (Schneider et al., 2019).

### Proteins

The investigation of the interplay between αSyn and proteins is of high relevance for understanding both the physiological and the pathological role of αSyn. To see how this interaction influences its monomeric structure, in the following paragraphs and in Table 2 we will summarize various biomolecules and how they affect not only its conformation but also its aggregation tendency.

**TABLE 2:** Reported proteins interactions with monomeric and aggregated states of αSyn.

Compound	α-Syn interaction region	α-Syn state conversion upon interaction	Common features (class)	References
Tubulin	Not specified	Folding into helical structure	Microtubule protein	Cartelli et al. (2016)
Hsp70	NAC and _95_VKKDQ_99_ (at the border between NAC and C-terminal)	Fibrils → soluble conformers	Chaperone	Ebrahimi-Fakhari et al. (2011)
		Monomers → stabilized monomers		Dedmon et al. (2005a)
				Luk et al. (2008)
				Klucken et al. (2006)
Hsp73	Not specified	Monomers → stabilized monomers	Chaperone	Chaari et al. (2016)
DNAJB6	Not specified	Non specified	Co-chaperone	Månsson et al. (2014)
				Aprile et al. (2017)
DNAJB1	Non specified	Fibrils → shorter fibrils → monomers	Co-chaperone	Gao et al. (2015)
SCGN	Non specified	Monomers and early-stage oligomers → soluble conformers	Ca^2+^-binding protein	Chidananda et al. (2019)
AS69	Y_39_, H_50_	Monomers → stabilized monomers	Engineered protein	Mirecka et al. (2014)
				Agerschou et al. (2019)

#### Tubulin

Tubulin is a highly conserved αβ dimeric protein that is the main component of microtubules. αβ-Tubulin dimers assembly and disassembly are finely tuned within the cell and a huge number of proteins interact with them, affecting the stability of microtubules and their function. Recently, it has been found that αSyn binds to microtubules and tubulin α_2_β_2_ tetramer. This interaction induces helical αSyn folding, enabling it to promote microtubule nucleation and to enhance microtubule growth rate and catastrophe frequency. On the other hand, PD αSyn mutants do not undergo tubulin-induced folding, causing tubulin aggregation rather than polymerization (Cartelli et al., 2016). However, the precise sequence of α-Syn binding site to tubulin has not been fully elucidated yet, and molecular studies aimed to deciphering the interaction at an atomic level are still missing.

#### Heat Shock Protein 70 (Hsp70) and Heat Shock Protein 73 (Hsp73)

**FIGURE 8:**
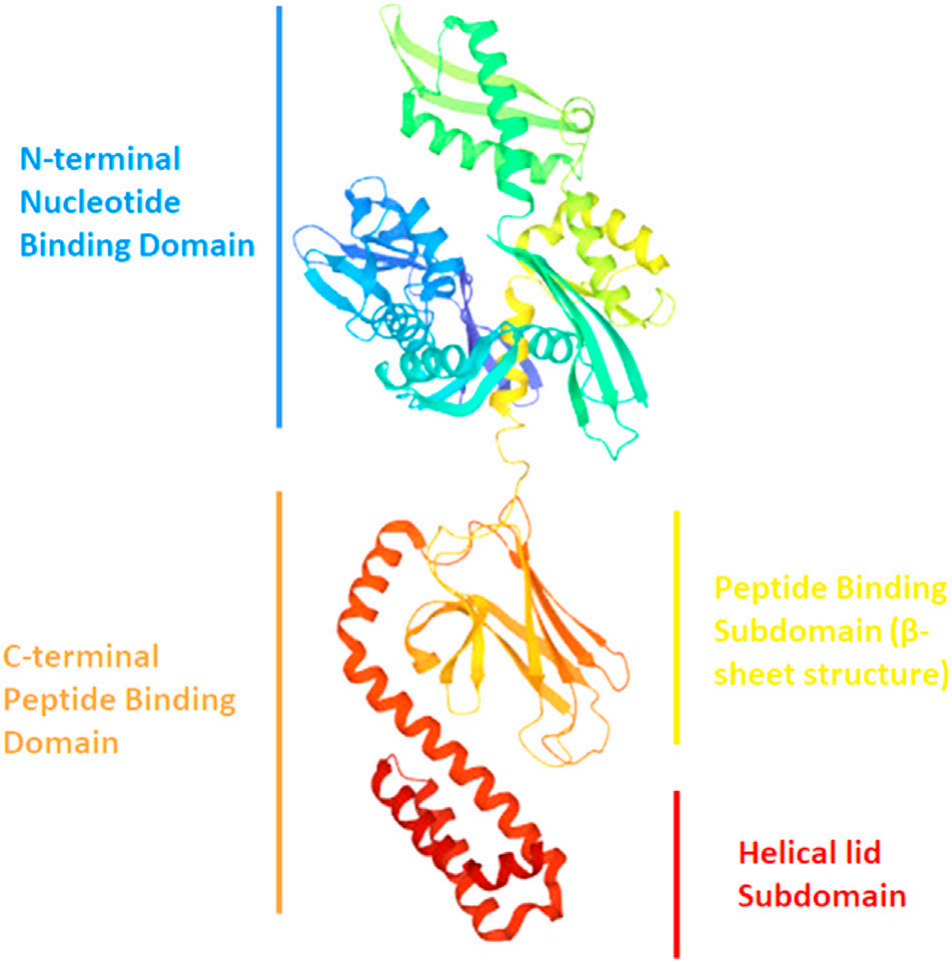
Ribbon drawing illustrating the structure of Hsp70 with its different domains (PDB file 2KHO).

Hsp70 is a 70 kDa protein from the “chaperone” family, involved in cell defense against protein misfolding (Figure 8). Concerning αSyn interaction, *in silico* and ThT assays suggest that Hsp70 binds to several conformers (monomers, protofibrils and fibrils) but it shows preference for the protofilaments involved in fibrils*.* In this regard, Fakhari et al. highlighted the binding of Hsp70 to the pre-fibrillar species of αSyn, which leads to their disassembly into soluble entities *in vitro* (Ebrahimi-Fakhari et al., 2011). Furthermore, experiments by Dedmon et al. show that Hsp70 interacts with αSyn fibrils instead of monomers. These results suggest that the protein adopts a folded structure which protects the central hydrophobic region and does not allow further inter-molecular binding. As shown by NMR data, this happens when the *C*-terminal domain makes contacts with the NAC region. If these interactions are perturbed (early stages of aggregation), the central region becomes exposed and this can lead to protein-protein interaction with the formation of pre-fibrillar aggregates. In this case, the chaperone binds to these aggregates and prevents the fibrils formation (Dedmon et al., 2005a). The hypothesis of Hsp70 interacting with the NAC region of αSyn is sustained also by Luk et al. As previously mentioned, this element represents the core of αSyn fibrils, and it contains the sequence required for αSyn to aggregate. Interestingly, ThT assay demonstrated that αSyn residues _95_VKKDQ_99_, at the border between NAC and the *C*-terminal domain, are crucial for interaction with Hsp70 (Luk et al., 2008).

Regarding monomers, Hsp70 is able to modify αSyn conformation by forcing it to a different open conformational state in which the *N*- and *C*-termini are distant from each other. αSyn-αSyn interactions are observed but are probably modified and NAC-NAC domain interactions among monomers are lost, increasing αSyn solubility (Klucken et al., 2006).

Another important chaperone is Hsp73 (Hsc70). Concerning its interaction with αSyn, Chaari et al. found out that the chaperone binds αSyn at the peptide binding sub domain (SBSD) corresponding to residues 386–509. These interactions involve unfolded monomers, and this is coherent with the role of Hsp73, which normally binds to unfolded proteins to mediate their refolding. At the same time, the helical subdomain (510–646) stabilizes the chaperone/αSyn complex, counteracting the formation of nuclei and/or the elongation of fibrils, as shown by *in vitro* experiments (Chaari et al., 2016).

#### DNAJB6 and DNAJB1

*DNAJB6*, the co-chaperone of Hsp70, is able to counteract αSyn and amyloid β aggregation *in vitro* by combining with its partner (Månsson et al., 2014). In particular, its effect is linked to the J domain, which catalyzes the transfer of the misfolded αSyn to the chaperone. Furthermore, *post-mortem* analysis on PD patients’ brains reveals the presence of the protein in Lewy Bodies, suggesting that its misregulation may provide early PD onset. Finally, this may reveal an interaction of DNAJB6 with αSyn and its direct role in aggregation inhibition. However, this hypothesis needs to be proved *in vivo* (Aprile et al., 2017). Focusing on the DNAJB family, *DNAJB1* efficiently works with Hsp70 and Hsp110 in fibrils disassembling. The system binds pre-formed fibrils both *in vitro* and *in vivo*, converting them into shorter fibrils later depolymerized into monomers (Gao et al., 2015). However, the interaction sites on αSyn still remain unknown.

#### Secretagogin (SCGN)

Studies suggest that neurodegeneration may be associated with Ca^2+^ dis-homeostasis, since a misregulation in this ion signaling system can be detected in neuropathologic patient brains. Considering this, scientists from Chidananda research team focused on SCGN, a Ca^2+^-sensor protein expressed in the brain which plays a key role in insulin regulation (Chidananda et al., 2019).

To study its effect over αSyn, the authors developed a method able to lead to protein fibrillation with entities of the range of 5–10 nm. Notably, TEM analysis revealed that no fibril was formed when αSyn was incubated with SCGN. These results are explained by considering that SCGN can bind both to monomers and early-stage oligomers, according to ThT and MTT (3-(4,5-dimethylthiazol-2-yl)-2,5-diphenyltetrazolium bromide) assays. In this case, soluble αSyn is preserved, without any further aggregation (Chidananda et al., 2019).

All in all, SCGN is shown to bind to αSyn and prevent it from fibrillation and nucleation *in vitro*. This may impede its binding to membranes, its misfolding and its aggregation. Finally, NMR studies show that anti-fibrillar activity is attributed to the central region and *C*-terminal domain of SCGN (Chidananda et al., 2019).

#### AS69

**FIGURE 9:**
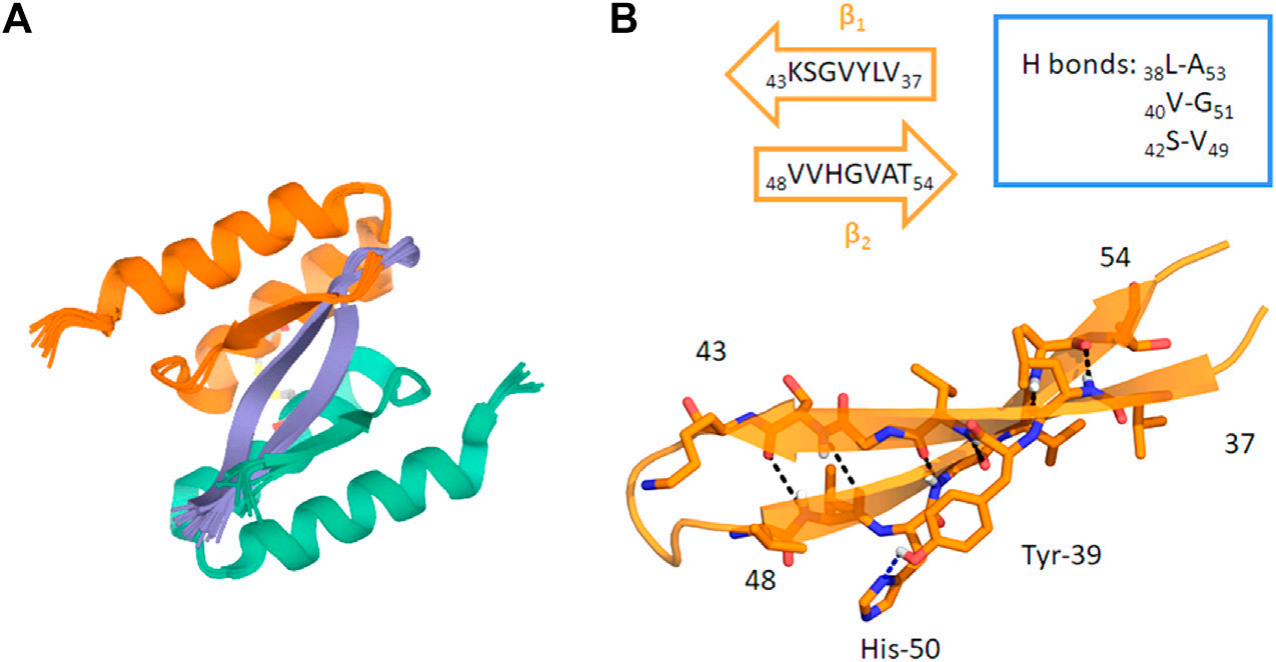
**(A)** Ribbon drawing of the AS69 structure interacting with the αSyn β-hairpin (residues 36–55). In light orange and green, the two subunits of AS69 are shown. Each subunit spans residues 13–58 of AS69. **(B)** Ribbon drawing of the αSyn β-hairpin (orange, β1 and β2 strands). H-bonds are depicted by dashed lines. The main interacting residues are shown as sticks (Mirecka et al., 2014).

AS69 was engineered from Mirecka et al., who developed a new phage library, obtained by random mutagenesis of the gene encoding ZAβ3. (Mirecka et al., 2014). This protein was proven to be an efficient Aβ_1–42_ aggregation inhibitor. In particular, due to its structure it is classified as “β-wrappin.” This protein shows two identical subunits, each formed by two α-helix and one β-strand spanning residues 13–58, linked by a disulphide bond involving the Cys28 residues of both of them. Moreover, NMR analyses showed that Phe31 residues of both AS69 subunits are involved in π-stacking interactions with Tyr39 and His50 of αSyn. Furthermore, molecular modeling studies suggest that AS69, by interacting with αSyn, folds into two β-strands and four α-helices forming a hydrophobic cavity where αSyn is buried (Figure 9) (Mirecka et al., 2014).

ThT analysis suggests that AS69 binds stoichiometrically to αSyn monomers, thus blocking the fibril elongation step by sequestrating free monomers. Also, the complex αSyn/protein can act as an inhibitor of the secondary nucleation process. Together, these results suggest that AS69 may display a broad activity against fibrillation, as demonstrated both *in vitro* and *in vivo* (*Drosophila* flies and mice) (Agerschou et al., 2019).

### Endogenous Small Molecules

**FIGURE 10:**
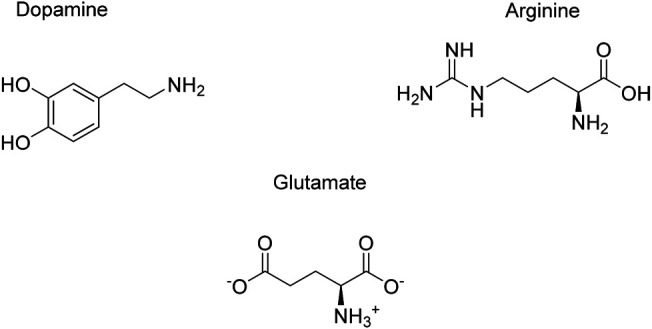
Endogenous small molecules affecting αSyn conformation and aggregation.

The role of endogenous small molecules (e.g., neurotransmitters) is important when it comes to understanding the function and structure of amyloids. Interestingly, some neurotransmitters are able to alter αSyn folding while interacting with it, which enables to better understand how the protein behaves and which binding sites are pivotal in that circumstance. The structures of the endogenous small molecules that will be reviewed in the next paragraphs are represented in Figure 10 and the effect are summarized in Table 3.

**TABLE 3:** Reported endogenous and exogenous small molecules interactions with monomeric and aggregated states of αSyn.

Compound	α-Syn interaction region	α-Syn state conversion upon interaction	Common features (class)	References
Dopamine	_125_YEMPS_129_ (C-terminal) and E_83_ (NAC)	Oligomers → complex/trimers lacking β-sheets	Neurotransmitter	Oliveri, (2019)
				Rekas et al. (2010)
				Post et al. (2018)
Arginine	Aromatic residues	Monomers → unstructured conformer	Neurotransmitter	S. Ghosh et al. (2018)
Glutamate	Not specified	Monomers → β-sheet rich oligomers	Neurotransmitter	S. Ghosh et al. (2018)
EGCG	Residues 23–55 (N-terminal)	Monomers and fibrils → non-toxic entities	Polyphenol	Xu et al. (2020)
				Caruana et al. (2011)
				Pujols et al. (2018)
				Sternke-Hoffmann et al. (2020)
Baicalein	Lysine and tyrosine side chains (mainly N-terminal)	Monomers and fibrils → non-toxic oligomers	Polyphenol	Kurnik et al. (2018)
				Morshedi et al. (2015)
				Oliveri, (2019)
				Javed and Ojha, (2020)
NDGA	V_3_, F_4_, Met_5_ and H_50_ (N-terminal)	Monomers → stabilized monomers	Polyphenol	Caruana et al. (2011)
		Fibrils → low β-sheet complexes		Daniels et al. (2019)
				Perni et al. (2017)
Squalamine	Residues 113–139 (C-terminal)	Not specified	Triterpenoid	Perni et al. (2017)
Nicotine	Not specified	Monomers → soluble oligomers	Alkaloid	Kardani et al. (2017)
Caffeine	Not specified	Oligomers → mature aggregates	Alkaloid	Oliveri, (2019)
				Kardani and Roy, (2015)
Mannitol	Not specified	Oligomers → non-toxic entities	Sugar alcohol	Shaltiel-Karyo et al. (2013)
Scyllo-inositol	NAC	Monomers → stabilized monomers	Sugar alcohol	Ibrahim and McLaurin, (2016)
TANI and TAN IIA	Not specified	Monomers/oligomers/fibrils → non-toxic entities	Phenanthrenequinone	Ji et al. (2016)
				Ren et al. (2017)
Cuminaldehyde	Lysine side chains (N-terminal)	Monomers → α-helix-like complexes	Aldehyde	Morshedi et al. (2015)
PcTs	F_4_ and Y_39_ (N-terminal), residues 93–95	Monomers → α-helix stabilized monomers	Phthalocyanines	Lamberto et al. (2009)
	(C-terminal)			Oliveri, (2019)
C41	N-terminal	Monomers/oligomers/fibrils → non-toxic entities	4-Hydroxynaphthalen-1-yl)sulphonamide derivatives	Kurnik et al. (2018)
NQTrp	Not specified	Monomers → non-toxic entities	Naphtoquinone-Tryptophan derivative	Paul et al. (2019)
M2N and M3N	Not specified	Fibrils → amorphic conformers	Mannitol derivatives	Paul et al. (2019)

#### Dopamine (DA)

Dopamine, one of our principal neurotransmitter, is a catecholamine implicated in several physiological process whose biosynthesis decreases in neuropathologies, like PD. Its role in αSyn aggregation modulation has been widely discussed and there is not a clear consensus whether it has a direct or indirect implication. In fact, some researchers hypothesize that DA can decrease αSyn fibrillation and oligomerization by binding to the protein *via* hydrophobic and hydrophilic interactions. These lead to non-stable complexes, which include its NAC or *C*-terminal region. Furthermore, studies showed that DA can mediate anti-fibrillar effect both *in vitro* and *in vivo*, while forming off-pathway oligomers (Oliveri, 2019).

The role of dopamine concerning αSyn modulation has been explored by Rekas et al. SAXS data suggest that the catecholamine mediates the formation of trimers made by αSyn overlapped structures. CD data suggest that their structure lacks β-sheets, which are crucial for amyloid aggregates (Rekas et al., 2010).

Recently, Post et al. reviewed the interaction between DA and αSyn, providing features over the protein structure and its binding sites. In particular, oxidized DA can interact with αSyn, producing a complex which enhances the formation of oligomers rather than fibrils (Post et al., 2018). Moreover, *in vitro* studies underline that its formation is due to a non-covalent binding between DA and the _125_YEMPS_129_ region of αSyn (Mazzulli et al., 2007). Furthermore, this complex seems to be stabilized by a salt-bridge between DA and E_83_ in the NAC region (Post et al., 2018).

Finally, the binding of DA to αSyn has an important effect on the conformation of the protein domains. In fact, fluorescence lifetime imaging microscopy data showed that the *N*- and *C*- termini of αSyn come closer, adopting a conformation which may inhibit fibril formation.

#### Arginine

Arginine is an amino acid able to affect αSyn behavior. This natural compound is well-known for its neuroprotective effect both *in vitro* and *in vivo* against glutamate excitotoxicity.

Regarding arginine/αSyn interaction, this molecule can inhibit protein late state aggregation, according to ThT, DLS and AFM (Atomic Force Microscopy) data. Isothermal calorimetry (ITC) and MS (Mass Spectrometry) analyses show that arginine binds to αSyn, forcing it to acquire a conformation thought to slow down the early-stage oligomerization. From a structural point of view, this conformer leads to a unified compaction of unfolded monomers. Since this intermediate is stabilized by clusters of arginine, the oligomerization and further fibrillation are avoided. Furthermore, MALDI-TOF mass spectrometry, ITC (Isothermal Titration Calorimetry) and MD analyses show that the aromatic residues of αSyn and the guanidine moiety of arginine interact *via* cation-π forces. Finally, arginine protective effect against αSyn toxicity was also proven in HeLa and SH-SY5Y cells line (S. Ghosh et al., 2018).

#### Glutamate

Glutamate is an excitatory neurotransmitter whose concentration in blood is around 50 µM. In the brain, it is the precursor of glutamine in presynaptic terminals and glial cells (Ghosh et al., 2018). Importantly, glutamate is shown to influence αSyn conformation and promote its aggregation. However, as an osmolyte, it tends not to directly interact with the protein; thus, its activity on αSyn may derive from its exclusion from the protein surface. The impact of glutamate on the conformation of αSyn is shown in *in vitro* assays. Interestingly, the more the concentration of glutamate is increased, the more unfolded monomers convert into β-sheet rich oligomers. In particular, small oligomers (10–15 nm diameter) predominate when glutamate is present at a concentration of less than 100 mM. Furthermore, AFM analysis proved that in glutamate treated samples, after 3 h of incubation two kinds of oligomeric aggregates appeared. The most representing one had a diameter of 20–35 nm, while the second one of 60–85 nm. Finally, this early stage oligomerization could be a critical factor to enhance fibrillation (Ghosh et al., 2018).

## Effect of Exogenous Factors on αSyn Structural Features

**FIGURE 11:**
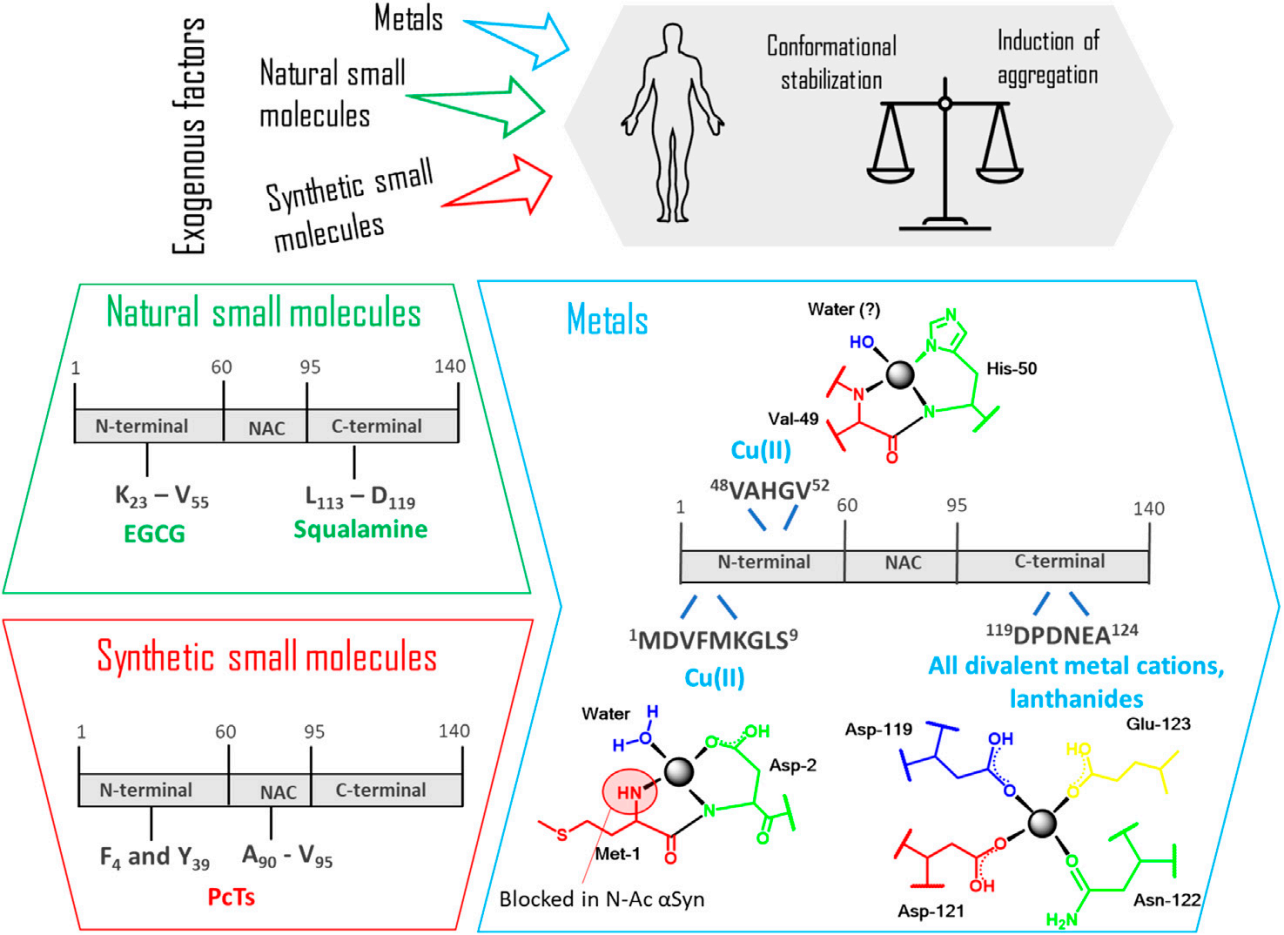
Binding sites recognized in the interaction between αSyn monomer and different exogenous factors (natural and synthetic small molecules, metals). The coordination between the protein and these factors leads to a balance that is always shifting between conformational stabilization and induction of aggregation. In the box regarding “metals,” the specific coordination modes of Cu(II), all divalent cations, lanthanides with the specific amino acid residues are detailed. EGCG: Epigallocatechin gallate; PcTs: phthalocyanine tetra sulfonate.

In general, the interaction of IDPs with exogenous compounds plays a crucial role for conformational stabilization and induction of aggregation. These chemicals can be found in the daily diet (e.g., flavonoids) or can derive from pharmacological treatments or habits (e.g., nicotine from smoking). Recognizing which elements are essential, beneficial or toxic is a very important topic, displaying each substance a bivalent effect as summed up by the sentence “The dose makes the poison”, asserted by Paracelsus. This is valid for every element, including metals, which have crucial physiological roles but at the same time can induce toxicity according to their therapeutic window. Thus, by analyzing the interaction between αSyn monomers and those molecules, a better insight of αSyn structural changes can be given. Finally, thanks to modern spectroscopy and molecular dynamics, the sites of interactions can be investigated. In the end, these data will help to better comprehend the structure of αSyn, whose details have not yet been fully elucidated. In this context, the main classes of chemicals that interact with αSyn are presented below (Figure 11).

### Metals

Metals are everywhere. These so-called trace elements, have an indispensable physiological role in normal brain functions, being often used by enzymes and proteins, due to their redox potential (Garza-Lombó et al., 2018). On the other hand, many recent epidemiological studies detected a significant higher level of metals in the affected brain regions of Parkinson's disease (PD) patients. In particular, high concentrations of iron, zinc and aluminum have been found in the *substantia nigra*, while copper accumulation has been detected in cerebrospinal fluid of PD patients. Furthermore, long-term metal exposure has been frequently related to parkinsonism (Bjørklund et al., 2019, 2020). Despite these numerous examples, there is still a controversial debate among experts whether metals are directly related to the cause of the disease. In general, we can state that the exact role of metals in the mechanism to neurodegeneration is still ambiguous. Indeed, it has been observed that metals catalyze the formation of reactive oxygen species causing oxidative stress but also enhance the aggregation of several proteins, among which αSyn, by complexing to them. At the same time, current studies have also shown how Mn and Ca levels can be regulated by αSyn itself (Dučić et al., 2015). But how can metals affect αSyn assembly? A possible explanation regarding the increased tendency to fibrillation is the subsequent conformational change after metal binding, resulting in abnormal folding and oligomer stabilization, as demonstrated for various metal ions (Kostka et al., 2008; Rcom-H’cheo-Gauthier et al., 2014; Uversky et al., 2001).

Regarding metal-protein complexation, many recent studies have been focused on determining the structural complexity of this interaction reaching some important milestones. A low affinity binding site exists at the *C*-terminus of αSyn, where carboxylates of Asp and Glu residues are the major contributors for metal binding. In particular residues 119–124 are involved in electrostatic interactions and can bind all divalent metal cations, without specificity. Additionally, the affinity to this binding site can be drastically increased after phosphorylation of Tyr-125 and Ser-129 as demonstrated by ESI-MS and fluorescence spectroscopy in the case of Cu(II), Fe(II) and Pb(II) (Lu et al., 2011).

In the hierarchical order of divalent metal cations binding to αSyn, copper has been recognized to be the most affine and efficacious metal in promoting aggregation. Its binding has peculiar features in comparison to other ions and an exhaustive structural description of its coordination to αSyn has been comprehensively summed up by Binolfi et al. (2012). The authors took into consideration the intrinsically disordered monomer of αSyn and recognized three different binding sites for Cu(II). Apart from the common *C*-terminal binding site, as previously described, two independent sites in the *N*-terminal portion have been defined as high-affinity binding site 1 (residues 1–5) and low-affinity binding site 2 (associated to His-50). Binding constants vary depending on the experimental conditions, so a comparison between results from different publications is not always appropriate. Anyhow, the authors could conclude that, differently from the binding to the *C*-terminal region typical of all the other cations, the *N*-terminal coordination might occur under physiological conditions and be significantly relevant to the beginning of PD.

After this review from 2012, many new experimental data have been published but still many questions remain open. In fact, current studies have partially undermined some of the previously described conclusions. In the following paragraphs, the influence of different metals on the αSyn structure will be analyzed and the most recent results in this field will be shown, taking into account contradictory point of views.

### Copper

A very important issue pointed out by the research group of Lucas in 2019 is the fact that αSyn is mainly present *in vivo* as a *N*-terminally acetylated protein (Abeyawardhane et al., 2019). It is immediately clear how this post-translational modification could have an important consequence in the copper-protein interaction, perturbing the high affinity *N*-terminal binding site, since the Met1 site is now blocked. Through electron paramagnetic resonance (EPR) spectroscopy based on the Peisach-Blumberg correlation diagram and the DFT calculations previously reported by Ramis et al., two new correlation modes have been described (Ramis et al., 2017). A N3O1 binding involving His50, Val49 and a water molecule has been identified as the preferential *N*-terminal binding site and the principal binding site of the *N*-terminal-acetylated αSyn. On the contrary, a *C*-terminal binding site including residues Asp119, Asp121 and Glu123 have a great impact on fibrillation of the H50Q missense mutation, enhancing the protein aggregation propensity. In a recent comparison study carried out by Lorentzon et al., Cu(II) was found to accelerate non acetylated αSyn aggregation at biologically-relevant metal ion concentrations, while this reaction was not affected at all in the presence of the acetylated protein, of the A53T mutant and of the 1–97 truncated version. This is probably correlated with the intrinsic aggregation speed of the various αSyn variants: since the velocity with which the variants form the amyloid is higher than that of the wild type, the effect of metal binding is not detectable anymore (Lorentzon et al., 2020).

Cu(I) has also been investigated even if less information has been generated about it. αSyn, in fact, interacts with both oxidation states of copper ions that are involved in a copper catalyzed oxidation reaction, with the subsequent formation of reactive oxygen species (ROS) that leads to oxidative stress and to a possible formation of amyloid fibrils (Bisaglia and Bubacco, 2020). Also, in the case of Cu(I), three binding sites have been recognized by NMR at the *N*- and *C*-termini, respectively residues 1–5 (high affinity), His-50 and residues 116–127 (Binolfi et al., 2011; Camponeschi et al., 2013; Miotto et al., 2014; Okita et al., 2017). In particular Met1 and Met5 are the main coordinating center for this ion with a 2S2N/O coordination mode (De Ricco et al., 2015).

### Iron

Also iron undergoes an oxidation cycle between two oxidative states Fe(II) and Fe(III) with production of ROS through the Fenton-Haber Weiss reaction (McDowall and Brown, 2016). Even in this case, as for copper, Lucas and coworkers investigated the influence of iron on the aggregation propensity and the secondary structure of the *N*-acetyl-αSyn (Abeyawardhane et al., 2018). Experiments performed in aerobic conditions showed that Fe(II) yielded a distinctive, highly toxic αSyn-metal complex in comparison to Fe(III). Fe(II), in fact, can react with O_2_ and oxidize to Fe(III) with the production of H_2_O_2_ and the subsequent development of a right-twisted antiparallel β-sheet conformation based on CD analyses and descriptive deconvolution of the secondary structure. These results display how the Fe(II) reactivity can have a very important impact in the protein conformation and its aggregated structural properties. Most importantly, the same does not occur with copper ions, proving a distinguished aggregation process.

### Calcium

Calcium dysregulation has been connected with neurodegenerative disorders and high levels of this metal have been detected in Lewy bodies. For its central role in αSyn aggregation, Kim and his research group took Ca^2+^ as representative metal ion to understand metal influence on the formation of large interfibrillar aggregates (Han et al., 2018). The authors could demonstrate that Ca^2+^ mediates the rapid formation of αSyn fibrils *via* the structural transition of monomeric αSyn into an extended conformation, which is prone to aggregation. It is interesting to discover how the structure of the α-syn monomer develops after binding to Ca^2+^. By using ion mobility-mass spectrometry (IM-MS) and synchrotron small-angle X-ray scattering (SAXS), Han et al. could demonstrate a structural transition of monomeric α-syn into an extended conformation with the exposure of the NAC region, which is more prone to aggregation.

### Lanthanide (Trivalent) Metal Ions

Investigation on lanthanides is a very crucial topic since they are increasingly applied in various fields of industry and agriculture. As divalent metal ions, trivalent metal ions non-specifically bind to the *C*-terminus of αSyn but also transiently interact with carboxylates in the N-terminal and NAC regions as interpreted from 1H to 15N HSQC NMR spectroscopy. In addition, they accelerate fibrillation much faster than divalent cations (Bai et al., 2015).

All the *in vitro* experiments carried out so far do not necessarily translate *in vivo* metal binding. Lothian et al. pointed out that there is a lack of evidence that the metal binding observed *in vitro* also occurs *in vivo* (Lothian et al., 2019). This work does not exclude the possibility that a very small percent (1%) of the whole protein can effectively bind to metals, promoting their aggregation with the consequent formation of oligomers and fibrils but, in general, according to the authors, αSyn cannot be considered as a metalloprotein *in vivo*. However, also these last results have some limitations because they considered non-pathogenic tissues, while in PD many factors can be combined and lead to the ultimately conclusion, like e. g post translational modification, molecular binding, ionic strength, salt concentration.

### Natural Small Molecules

Natural products are gaining importance in drug discovery since they are an environmental-friendly source for hit compounds. Moreover, with modern extraction and purification techniques, researchers are able to obtain these small molecules with moderate efforts. Also, they offer low-cost production and possible improvement of their activity. However, natural compounds have some limitations such as low reproducibility and yield, but also lack of safety and tolerability. Finally, their multi-target activity can be a problem when the aim is to be selective toward a single target.

**FIGURE 12:**
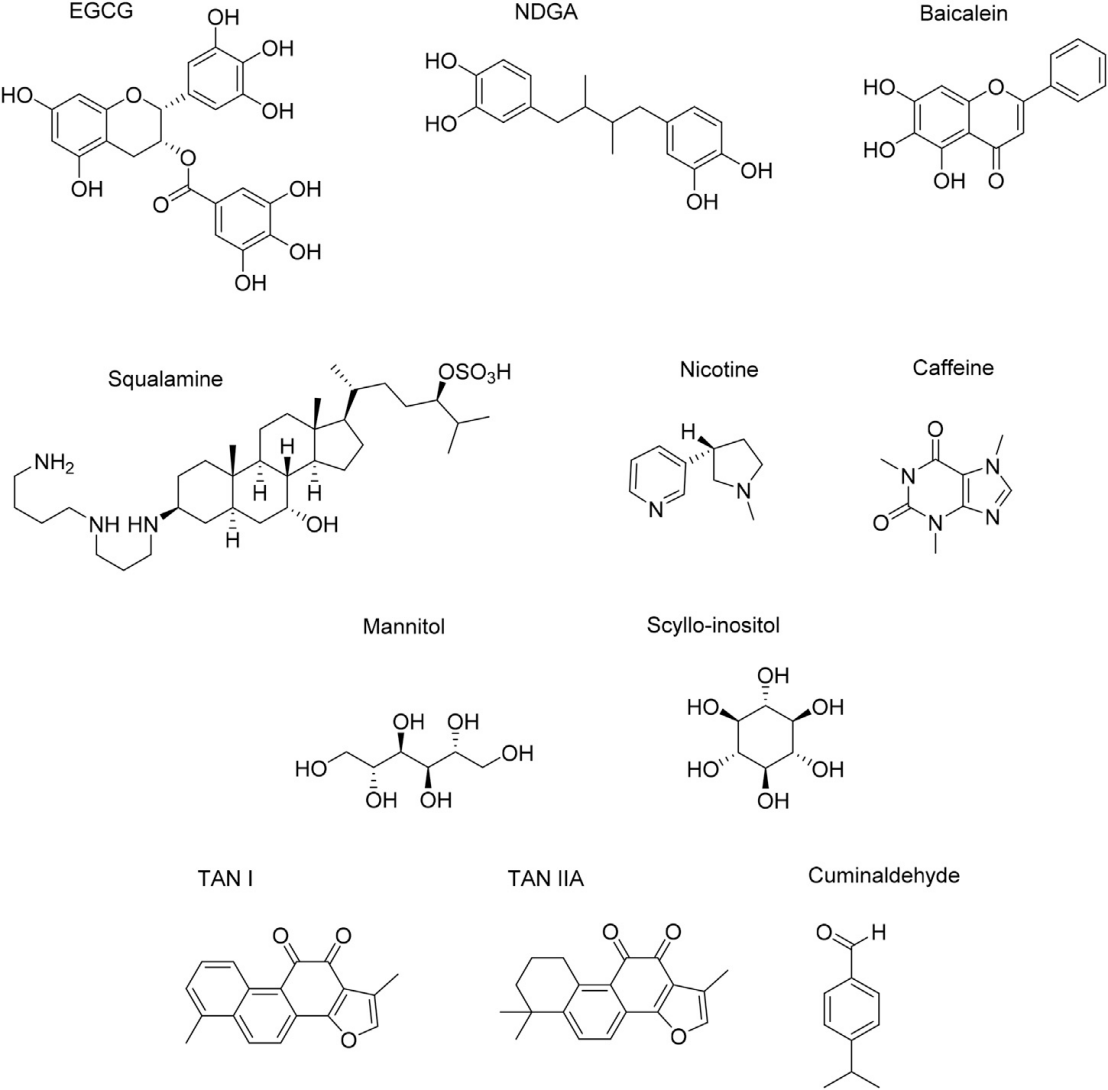
Natural small molecules influencing αSyn conformation properties.

Since these molecules present low selectivity, they can bind to both αSyn aggregates and monomers. However, when they bind to monomers, few of them have a characteristic binding site, hence more studies are needed to look further into this topic. Here, we present an overview over the main classes of natural small molecules able to influence monomeric αSyn aggregation. Their structures are depicted in Figure 12.

### Polyphenols

#### Flavonoids

##### Epigallocatechin Gallate (EGCG)

EGCG is a natural compound known for its antioxidant properties and anti-aggregation activity against amyloid proteins. This latter effect against multiple targets (αSyn, Aβ_1–42_, Tau, hIAPP) is due to its lack of selectivity. Concerning αSyn, NMR studies suggest that EGCG binds to the *N*-terminal domain, in particular to residues 23–55 (Xu et al., 2020). This binding is mainly governed by Van der Waals and π-stacking interactions due to the structure of EGCG, characterized by electron-rich aromatic rings bearing three consecutive OH substituents. Even if the mechanism behind the anti-aggregation effect is ambiguous, EGCG can bind fibrils and convert them into smaller, non-toxic aggregates. Moreover, EGCG is also able to bind to monomers and induce their aggregations into non-cytotoxic, off-pathway entities, thus avoiding the nucleation process (Caruana et al., 2011). EGCG efficacy has been tested both *in vitro* and *in vivo* (Pujols et al., 2018). Also, the compound is currently under clinical trials for Multiple System Atrophy (Xu et al., 2020). Recent studies have reported that the species responsible for EGCG anti-aggregating properties is its oxidized form (oxEGCG). In fact, most of the EGCG efficacy studies were performed at pH 7, at which the compound is not stable and comes across oxidation. When EGCG is tested at pH six or less, the molecule is stable and its anti-aggregation properties are lost (Sternke-Hoffmann et al., 2020).

##### Baicalein

Baicalein is a flavonoid extracted from *Scutellaria* baicalensis. This molecule is known to disassemble αSyn fibrils into smaller, non-toxic oligomers by binding to them once they are mature (Kurnik et al., 2018). As EGCG, baicalein is also active toward αSyn monomers: the polyphenol can interact and convert them into off-pathway aggregates with very low cellular toxicity (Morshedi et al., 2015).

Recently, Javed et al. reviewed the interaction between baicalein and αSyn. Here, the oxidized form of baicalein (quinone) is crucial for αSyn aggregation inhibition. In fact, its effectiveness against αSyn aggregation has been tested both in cells (HeLa and SH-SY5Y) and *in vivo* models (Oliveri, 2019). When baicalein quinones interact with early-stage aggregates, it leads to quite soluble αSyn oligomers. In this case, the polyphenol covalently binds to the protein and creates a Schiff base with lysine side chains, expressed in the *N*-terminal domain of αSyn. Tyrosine residues are also involved in this binding (Javed and Ojha, 2020).

As mentioned before, Baicalein binds to a broad region of αSyn, thus it is not selective toward a specific binding site. Indeed, other studies showed that baicalein is an efficient aggregation inhibitor also for Aβ, Tau, IAPP and other amyloid proteins, which is a common characteristic for polyphenols (Oliveri, 2019).

##### Nordihydroguaiaretic Acid (NDGA)

Nordihydroguaiaretic acid is a natural compound deriving from *Larrea tridentata.* Concerning its interaction with αSyn, confocal single-molecule fluorescence spectroscopy studies showed that the compound binds to toxic inclusions and that it is able to inhibit the formation and/or to disaggregate mature oligomers (Caruana et al., 2011). Moreover, recent studies pointed out that oxidation and consecutive cyclization of NDGA is required for its activity. In fact, its oxidized form (oxNDGA) can interact with monomers and convert them into quinone-modified species, which are less prone to aggregate in comparison to the non-modified ones. However, these monomers can still carry out their physiological function: probably this modification does not alter their normal activity. In fact, as CD data suggest, monomers preserve the ability to fold into α-helix conformations while interacting with SDS *in vitro.* Also, in the same work, oxNDGA was shown to inhibit *in vivo* oligomers and fibrils formation (*C. elegans*) (Daniels et al., 2019).

Furthermore, the compound is also able to interact with preformed fibrils. In fact, ThT assays displayed that cyclized NDGA can reduce the contents of β-sheets in a dose-dependent manner (Perni et al., 2017). However, it is important to inquire if these interactions have an effect over αSyn structure. In particular, ESI-MS analysis showed no covalent binding, which means that the protein primary structure is unmodified. Moreover, this interaction leads to a more compact conformation of the protein, which may mask the NAC region and discourage aggregation. Nevertheless, this hypothesis still needs additional analyses to be confirmed.

Regarding the sequences of αSyn involved in this interaction, NMR studies suggested that the most engaged domain is the *N*-terminus (Val3, Phe4, Met5 and His50 in particular). Especially, this domain is involved in the monomer’s helix folding while interacting with membranes: as we saw before, the dynamic flexibility of the protein is not altered by the interaction with oxNDGA.

All in all, these results show that NDGA and in particular oxNDGA can modify the conformation of the protein aggregates. However, the structural properties of these entities as well as the involved domains of αSyn have yet to be elucidated.

### Non polyphenols

#### Triterpenoids

##### Squalamine

Squalamine is a steroid-polyamine conjugate found in sharks and is known for its anticancer and antiviral activity. Its main characteristic is the polyamine chain attached to the cyclopentanoperhydrophenanthrene structure, which is positively charged in the cell’s physiological environment. This moiety allows the compound to interact with membranes, in particular with the phospholipids negatively charged heads (Perni et al., 2017). Finally, the salt bridges formed between squalamine and the lipidic bilayer may avoid the pathological formation of αSyn oligomers, since there is a competition for the same sites of interactions between misfolded αSyn monomers and the triterpenoid. These hypotheses were confirmed by CD experiments. In fact, when αSyn is incubated with squalamine in the presence of phospholipidic membranes, a decrease of the α-helix character is noticed. Thus, one can speculate that the displacement of helical-folded αSyn from membranes leads to the refolding of the protein in a random-coil configuration. This last structure is the one in which monomers are usually found and it represents the most populated state of soluble cytosolic αSyn. However, further studies are required to confirm this idea.

Concerning αSyn-squalamine interactions, NMR analyses showed that the *C*-terminal domain is the most involved region of the protein (Perni et al., 2017). This is consistent with the fact that the positively charged chain of squalamine can create electrostatic interactions with the negatively charged domain of αSyn. In particular, the sequence engaged spans residues 113–139. However, this interaction is attenuated when squalamine and αSyn are incubated in the presence of membranes. Furthermore, this contact does not seem to directly alter the conformation of the protein. In fact, squalamine prefers to interact with phospholipids instead of αSyn in the cells. Nevertheless, more studies are required to confirm this speculation to be sure that αSyn refolding merely refers to its displacement by membranes.

Finally, the prevention of toxic oligomers formation provided by squalamine was proven *in vitro,* and the antiaggregant properties were later tested and confirmed *in vivo* using a *Caernohabditis elegans* PD model (Perni et al., 2017).

#### Alkaloids

##### Nicotine and Caffeine

Nicotine and caffeine can interact with αSyn and inhibit its aggregation pathway. However, since they can bind to αSyn simultaneously, their respective binding sites may be different. Also, the mechanism of the inhibition of aggregation is still unclear.

Concerning nicotine, *in vitro* studies demonstrated that this alkaloid can induce a conformational change in αSyn monomers, leading to nucleation slowdown and the formation of soluble, less toxic oligomers (Kardani et al., 2017).

At the same time, caffeine can decrease αSyn aggregates toxicity, while accelerating the apparent fibrillation rate (Oliveri, 2019). Interestingly, CD and TEM data suggest that caffeine does not alter the conformation of αSyn monomers (Kardani and Roy, 2015). Also, an increased transformation of oligomers into mature aggregates by administration of caffeine in yeast cell is remarked in literature. These data suggest a role of this alkaloid in the field of synucleinopathologies (Kardani and Roy, 2015).

Finally, nicotine is proven to be active against Aβ aggregates, while caffeine displays an activity also toward hIAPP and Tau toxic species both *in vitro* and *in vivo* (Ma et al., 2020).

#### Sugar Alcohols

##### Mannitol

Mannitol is a polyol approved by the Food and Drug Administration as an osmotic agent. Also, it is used as an osmotic diuretic in the therapy of hypertension and as a weak laxative in case of constipation. Furthermore, mannitol is known for its BBB disruption activity and its hyperosmotic solution is widely used in clinics. CD studies show that this compound is able to inhibit the aggregation of αSyn monomers into fibrils, likely through interaction with oligomers by leading them to an alternative pattern of aggregation acting as a “chemical chaperone”. Finally, its neuroprotection activity was tested and proven *in vivo* in *Drosophila* flies and in mice (Shaltiel-Karyo et al., 2013).

Focusing on αSyn-mannitol interaction, CD experiments show that the polyalcohol does not affect the conformation of β-sheet rich fibrils, so no interaction is detected with the mature aggregates of the protein. However, the compound is able to change the secondary structure of αSyn oligomers. In fact, CD analysis spots a refolding in the early-stage aggregates outline. However, more studies are required to understand the structural properties of the entities derived by oligomers refolding. Also, the protein domains involved in this interaction have yet to be discovered (Shaltiel-Karyo et al., 2013).

##### Scyllo-Inositol

Scyllo-inositol is one of the inositol stereoisomers, rare in nature, having attracted the attention of the scientific community in the field of Aβ_1–42_ peptide inhibition. In fact, scyllo-inositol was shown to stabilize a non-toxic form of Aβ_1–42_ peptide and to ameliorate cognitive deficit together with lowering amyloid plaques *in vivo* (AD mouse model) (Ibrahim and McLaurin, 2016). Interestingly, this molecule was proven to have an effect also toward αSyn. In fact, TEM experiments suggest that it can reduce both human and mouse αSyn aggregation. An explanation for its activity may lay in its planar structure, which is expected to interact with αSyn monomers through hydrophobic and hydrophilic interactions, possibly entrapping the NAC domain of the protein. Since this condition is crucial in fibrillation, its inaccessibility can discourage protein–protein interactions and, finally, aggregation. Considering αSyn conformation upon the interaction with scyllo-inositol, soluble monomers seem to be stabilized *in vitro*. This may allow these species to conserve their random coil structure and prevent them from the nucleation phase. However, further analyses are needed to determine the binding sites involved and the monomers behavior (Ibrahim and McLaurin, 2016).

#### Others

##### Tanshinone I (TAN I) and Tanshinone IIA (TAN IIA)

TAN I and TAN IIA are the main phenanthrenequinone compounds found in *Salvia miltiorrhiza,* a plant widely studied in Chinese traditional medicine. Concerning the interactions between these compounds and αSyn, ThT and TEM studies suggest that they both prolong the lag time of αSyn aggregation and disaggregate mature fibrils (Ji et al., 2016). This effect is related to their role in decreasing toxic oligomers formation, which contributes to their multi-target activity. Moreover, they seem to interact with αSyn monomers and oligomers through hydrophobic interactions, blocking them from aggregation in the same way they do with Aβ_1–42_ peptide (Ren et al., 2017).

Regarding αSyn conformational aspects, CD data show that TANI and TANIIA keep the protein in a random coil structure, while in their absence monomers tend to misfold and aggregate in β-sheet structures (Ren et al., 2017). Although evidence suggests that the two compounds can avoid αSyn nucleation and fibrillation, the binding sites as well as a detailed description of the complex formed should still be investigated.

##### Cuminaldehyde

Cuminaldehyde is an aldehydic compound present in *Cuminum cyminum* essential oil. It is thought that the molecule interacts with αSyn monomers, thus preventing them from nucleation. Furthermore, cuminaldehyde showed a lower activity in fibrillar disaggregation than baicalein. These data suggest that Cuminaldehyde is more selective toward monomers rather than aggregated species (Morshedi et al., 2015).

Interestingly, far-UV CD gives an interesting insight over the structural behavior of αSyn while interacting with cuminaldehyde. When cuminaldehyde is incubated with αSyn, the strong negative peak at 200 nm disappears, in favor of one at 208 nm. This result highlights the conversion of random-coil monomers into entities whose structure is still unknow. However, no β-sheet peaks are detected and the shape of the graph refers to a characteristic α-helical conformation. All in all, one can speculate that the new complexes may adopt a helical structure, which is not prone to fold into β-sheet nor to convert into unfolded coils (Morshedi et al., 2015).

Interestingly, NMR studies suggest that the aldehydic function of cuminaldehyde may interact with lysine amino groups in the *N*-terminal domain of αSyn monomers (Morshedi et al., 2015). Thus, this interaction can be one of the main cause which leads to the conformational transition occurred in αSyn interacting with the compound. Finally, it may provide details about the binding site of the protein.

### Synthetic Small Molecules

**FIGURE 13:**
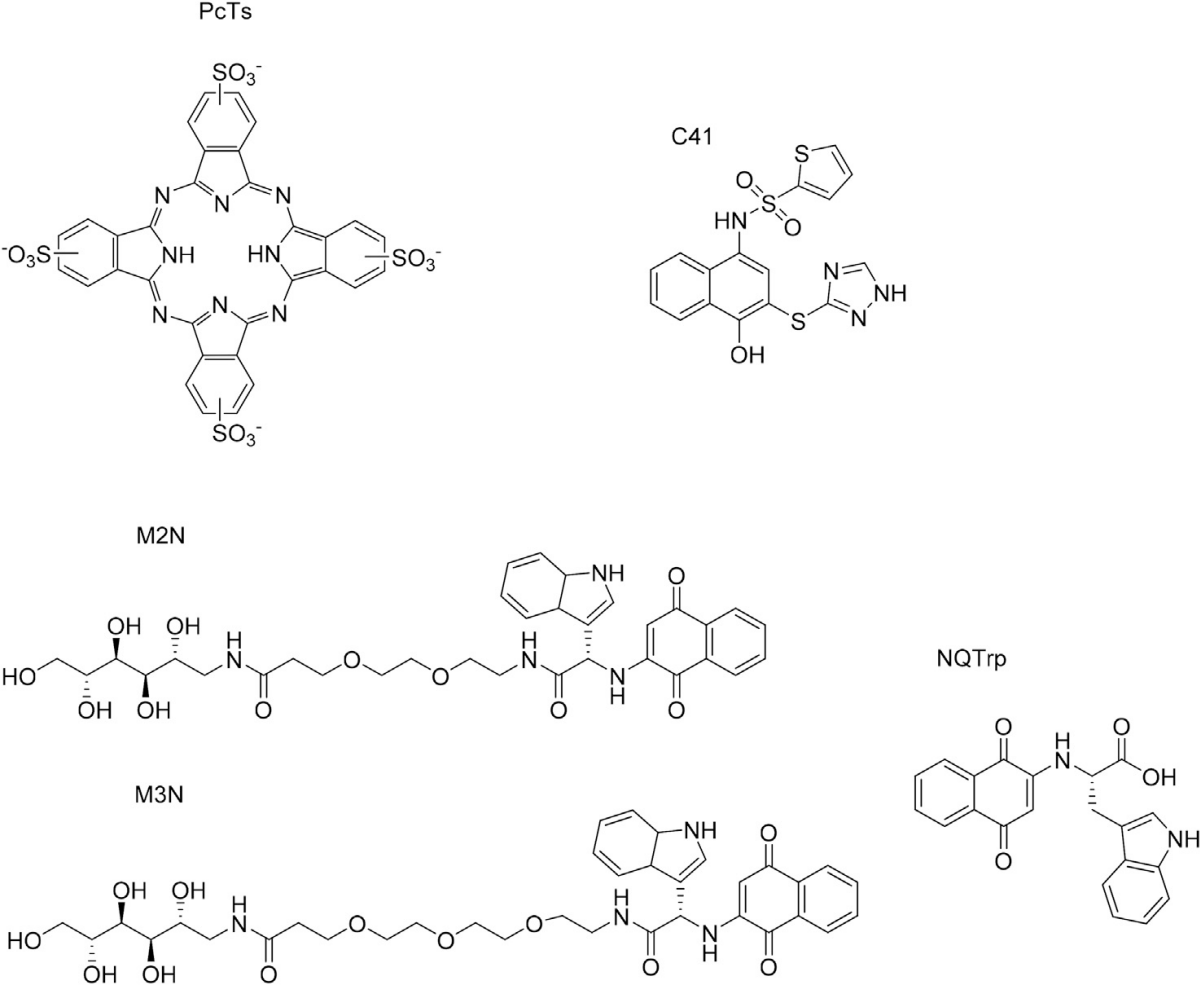
Synthetic small molecules effective on αSyn aggregation.

Synthetic small molecules have been widely investigated as putative inhibitors of αSyn aggregation. Here, some of the principal compounds currently being studied are described, highlighting the interaction with the protein (Figure 13). Other important molecules that play a role as inhibitors are omitted, since they mainly interact with αSyn aggregates rather than monomers. An example is the pyrazole Anle 138, widely described in Fields and Shvadchak works (Shvadchak et al., 2018; Fields et al., 2019).

### Phthalocyanines

Phthalocyanines are tetrapyrrole macrocycles largely investigated in the field of αSyn aggregation. Their structure is correlated to the mechanism by which they bind to the protein: both the electron-dense pyrrolic core and the substituent carried by the peripherical rings play a pivotal role. In fact, NMR studies show that phthalocyanine tetra sulfonate (**PcTs**, a synthetic derivative of this group) interacts with αSyn at the *N*-terminal region residues Phe4 and Tyr39 mainly by π–π stacking interactions and salt bridges. This leads to a stabilization of the α-helical folding of monomers, thus delaying their misfolding and aggregation. However, data about the binding mode of PcTs to αSyn are controversial. In contrast with the previous studies, high resolution ^1^H–^15^N HSQC-NMR data of Lamberto et al. demonstrated that the compound binds to the *C*-terminal domain of αSyn monomers. Thus, these studies suggest the existence of another important binding site, involving residues 93–95 of the protein (Lamberto et al., 2009). Considering this contradiction, further studies are needed to understand which site plays a role in the interaction between αSyn and phthalocyanines.

Interestingly, PcTs can form a complex with Cu^2+^, an important ion for αSyn accumulation in tissues. Also, this compound is able to inhibit fibrillation by forming off-pathway non-toxic oligomers *in vitro* (Oliveri, 2019). Finally, the compound is active not only against αSyn, but also amyloid β, Tau and PrP protein aggregates *in vitro* (Valiente-Gabioud et al., 2016).

#### 4-Hydroxynaphthalen-1-yl)sulphonamide Derivatives

These compounds are novel inhibitors revealed by High-Throughput Screening (HTS). Among them, one of the most active compounds is **C41**. *In vitro* studies show that this molecule binds to αSyn monomers, on-pathway oligomers, and fibrillary precursors. In particular, the interaction with soluble monomers was confirmed by Size Exclusion Chromatography–Multi Angle Light Scattering (SEC–MALS). Furthermore, C41 also binds to off-pathway small aggregates and this can prevent both vesicle interaction and nucleation. ^1^H–^15^N HSQC analysis demonstrated that C41 mainly interacts with αSyn *N*-terminal domain through hydrophobic forces. As we saw before, this domain is involved in αSyn interaction with membrane, which is crucial for αSyn physiological role. MS data show that covalent adducts can be formed, but more studies are needed to identify them and understand the conformational changes of αSyn (Kurnik et al., 2018).

### D3.3 Mannitol Derivatives

#### M2N and M3N

These compounds are αSyn aggregation inhibitors that consist of mannitol, covalently linked to NQTrp via two or three molecules of PEG. **NQTrp**, a generic amyloids inhibitor formed by NQ (Naphtoquinone) and Trp (Tryptophan), is effective against fibrils formation due to the possibility to share π-π interactions with αSyn monomers. However, even if the inhibition occurs at low concentration (0.1 µm), the compound is characterized by a poor BBB penetration (Paul et al., 2019).

To overcome this problem, researchers conjugated it with mannitol, known for its BBB disruption properties and anti- αSyn aggregation effect. Notably, the conjugates were non-toxic to SH-SY5Y cells and could reduce the cytotoxicity of αSyn aggregates. Moreover, results suggest that the longer PEG chain in M3N might confer better flexibility for a more efficient inhibition.

Concerning αSyn conformational aspects, CD studies were performed to elucidate the protein behavior during the interaction with the compounds. In this analysis, αSyn alone shows a negative peak at 218 nm and a positive one around 198 nm, which are typical of β-sheet rich structures. By adding incremental doses of the M2N and M3N, the peak at 218 decreases in a dose-dependent manner. This means that the secondary structure of the protein refolds during the interaction with the molecules, with a decrement of β-sheets. However, more studies are needed to identify the conformation of the new formed complexes (Paul et al., 2019).

Finally, TEM studies show that in the presence of M2N and M3N, the fibrillar outline of αSyn inclusions turns into an amorphic conformation (Paul et al., 2019). This is in accordance with the previous results and suggests that these inhibitors can be interesting to investigate αSyn behavior while interacting with polyalcohol compounds.

## Conclusion and Future Perspective

After this trip around αSyn structure, the factors influencing it and the applied techniques, it is clear that the fundamental structural features of this rather small protein of 140 residues have not yet been elucidated. The main hurdle to thoroughly understand its behavior is its intrinsically disordered nature and high susceptibility to the environment. αSyn tends to acquire diverse transient and dynamic conformations depending on the presence of different biological and physico-chemical factors. In physiological conditions, αSyn is thought to be a compact monomer acquiring an aggregation-resistant globular structure. This conformation is stabilized by long-range electrostatic interactions between the residues present in the *C*-terminal domain and those located in the central part of the protein. On the other hand, when αSyn is driven to adopt a more extended structure, exposing the NAC region, protein aggregation is triggered. It is assumed that folded stable helical conformers impede amyloidogenic aggregation. However, to date, there is not a clear consensus on its *in vivo* structural propensity. αSyn will likely adopt specialized conformations depending on different conditions (e.g., changes in pH, temperature, ionic strength, closeness to surfaces) that might trigger different biological or pathological functions. For instance, a helical pattern at the *N*-terminal end has been observed upon vesicle and membrane interaction. Conversely, there are limited comprehensive structural data about αSyn interactions with various partners such as proteins and both endogenous (e.g., neurotransmitters, and lipids) and exogenous (e.g., metals and drugs) molecules. This information is limited due to the difficulty to create, isolate and analyze complexes of αSyn with these partners. This lack of comprehensive knowledge is also due to the absence of crystallographic data and of other experimental techniques able to reproduce the *in vivo* physiological and/or pathological conditions. In addition, experimental data in the literature are obtained from the study of αSyn in very different conditions, hampering a significant comparison of the obtained results. In our opinion, it would be appropriate for studies to converge upon standardized set up and protocols. As an example, *N*-terminal acetylation has been demonstrated to be a constitutive element of the protein, so this modification should be applied in every experiment to achieve reliable conclusions. Other aspects to be considered are the source of αSyn, its post-translational modifications and pathological mutations. Finally, a combination of experimental and computational approaches can be a good strategy for future research. By extracting information from different experimental techniques and constraining molecular dynamic simulations based on that information, more meaningful results could be obtained, allowing us to address some of the experimental issues observed so far. The more reliable information obtained, the more effective their translation into the development of bioactive compounds able to modulate pathological αSyn effects will be.

## References

[B1] Abeyawardhane D. L. Fernández R. D. Murgas C. J. Heitger D. R. Forney A. K. Crozier M. K. (2018). Iron Redox Chemistry Promotes Antiparallel Oligomerization of α-Synuclein. J. Am. Chem. Soc. 140 (15), 5028–5032. 10.1021/jacs.8b02013 29608844

[B2] Abeyawardhane D. L. Heitger D. R. Fernández R. D. Forney A. K. Lucas H. R. (2019). C-terminal CuII Coordination to α-Synuclein Enhances Aggregation. ACS Chem. Neurosci. 10 (3), 1402–1410. 10.1021/acschemneuro.8b00448 30384594

[B3] Agerschou E. D. Saridaki T. Flagmeier P. Galvagnion C. Komnig D. Nagpal A. (2019). An Engineered Monomer Binding-Protein Forα-Synuclein Efficiently Inhibits the Proliferation of Amyloid Fibrils. Elife 8, e46112. 10.7554/eLife.46112 31389332 PMC6721797

[B4] Allison J. R. Varnai P. Dobson C. M. Vendruscolo M. (2009). Determination of the Free Energy Landscape of α-Synuclein Using Spin Label Nuclear Magnetic Resonance Measurements. J. Am. Chem. Soc. 131 (51), 18314–18326. 10.1021/ja904716h 20028147

[B5] Appel-Cresswell S. Vilarino-Guell C. Encarnacion M. Sherman H. Yu I. Shah B. (2013). Alpha-synuclein p.H50Q, a Novel Pathogenic Mutation for Parkinson’s Disease. Mov Disord. 28 (6), 811–813. 10.1002/mds.25421 23457019

[B6] Aprile F. A. Arosio P. Fusco G. Chen S. W. Kumita J. R. Dhulesia A. (2017). Inhibition of α-Synuclein Fibril Elongation by Hsp70 Is Governed by a Kinetic Binding Competition between α-Synuclein Species. Biochemistry 56 (9), 1177–1180. 10.1021/acs.biochem.6b01178 28230968

[B7] Bai J. Zhang Z. Liu M. Li C. (2015). α-synuclein-lanthanide Metal Ions Interaction: Binding Sites, Conformation and Fibrillation. BMC Biophys. 9 (1), 1–10. 10.1186/s13628-016-0026-1 26843956 PMC4739322

[B8] Balesh D. Ramjan Z. Floriano W. B. (2011). Unfolded Annealing Molecular Dynamics Conformers for Wild-type and Disease-Associated Variants of Alpha-Synuclein Show No Propensity for Beta-Sheetformation. Jbpc 02 (2), 124–134. 10.4236/jbpc.2011.22015

[B9] Bartels T. Kim N. C. Luth E. S. Selkoe D. J. (2014). N-Alpha-Acetylation of α-Synuclein Increases its Helical Folding Propensity, GM1 Binding Specificity and Resistance to Aggregation. PLoS ONE 9 (7), e103727. 10.1371/journal.pone.0103727 25075858 PMC4116227

[B10] Bédard L. Lefèvre T. Morin-Michaud É. Auger M. (2014). Besides Fibrillization: Putative Role of the Peptide Fragment 71-82 on the Structural and Assembly Behavior of α-Synuclein. Biochemistry 53 (41), 6463–6472. 10.1021/bi5008707 25255476

[B11] Bertoncini C. W. Jung Y.-S. Fernandez C. O. Hoyer W. Griesinger C. Jovin T. M. (2005). From the Cover: Release of Long-Range Tertiary Interactions Potentiates Aggregation of Natively Unstructured -synuclein. Proc. Natl. Acad. Sci. 102 (5), 1430–1435. 10.1073/pnas.0407146102 15671169 PMC547830

[B12] Bhattacharya S. Xu L. Thompson D. (2019). Molecular Simulations Reveal Terminal Group Mediated Stabilization of Helical Conformers in Both Amyloid-β42 and α-Synuclein. ACS Chem. Neurosci. 10 (6), 2830–2842. 10.1021/acschemneuro.9b00053 30917651

[B13] Binolfi A. Valiente-Gabioud A. A. Duran R. Zweckstetter M. Griesinger C. Fernandez C. O. (2011). Exploring the Structural Details of Cu(I) Binding to α-Synuclein by NMR Spectroscopy. J. Am. Chem. Soc. 133 (2), 194–196. 10.1021/ja107842f 21158432

[B14] Binolfi A. Quintanar L. Bertoncini C. W. Griesinger C. Fernández C. O. (2012). Bioinorganic Chemistry of Copper Coordination to Alpha-Synuclein: Relevance to Parkinson’s Disease. Coord. Chem. Rev. 256 (19–20), 2188–2201. 10.1016/j.ccr.2012.05.004

[B15] Bisaglia M. Bubacco L. (2020). Copper Ions and Parkinson’s Disease: Why Is Homeostasis So Relevant? Biomolecules 10 (2), 195. 10.3390/biom10020195 PMC7072482 32013126

[B16] Bjørklund G. Hofer T. Nurchi V. M. Aaseth J. (2019). Iron and Other Metals in the Pathogenesis of Parkinson’s Disease: Toxic Effects and Possible Detoxification. J. Inorg. Biochem. 199, 110717. 10.1016/j.jinorgbio.2019.110717 31369907

[B17] Bjørklund G. Dadar M. Chirumbolo S. Aaseth J. (2020). The Role of Xenobiotics and Trace Metals in Parkinson’s Disease. Mol. Neurobiol. 57 (3), 1405–1417. 10.1007/s12035-019-01832-1 31754997

[B18] Bodner C. R. Dobson C. M. Bax A. (2009). Multiple Tight Phospholipid-Binding Modes of α-Synuclein Revealed by Solution NMR Spectroscopy. J. Mol. Biol. 390 (4), 775–790. 10.1016/j.jmb.2009.05.066 19481095 PMC2709488

[B19] Bodner C. R. Maltsev A. S. Dobson C. M. Bax A. (2010). Differential Phospholipid Binding of α-Synuclein Variants Implicated in Parkinson’s Disease Revealed by Solution NMR Spectroscopy. Biochemistry 49 (5), 862–871. 10.1021/bi901723p 20041693 PMC2815556

[B20] Brodie N. I. Popov K. I. Petrotchenko E. V. Dokholyan N. V. Borchers C. H. (2019). Conformational Ensemble of Native α-synuclein in Solution as Determined by Short-Distance Crosslinking Constraint-Guided Discrete Molecular Dynamics Simulations. Plos Comput. Biol. 15 (3), e1006859–21. 10.1371/journal.pcbi.1006859 30917118 PMC6453469

[B21] Burré J. (2015). The Synaptic Function of α-Synuclein. Jpd 5 (4), 699–713. 10.3233/JPD-150642 26407041 PMC4927875

[B22] Bussell R. Eliezer D. (2001). Residual Structure and Dynamics in Parkinson’s Disease-Associated Mutants of α-Synuclein. J. Biol. Chem. 276 (49), 45996–46003. 10.1074/jbc.M106777200 11590151

[B23] Camponeschi F. Valensin D. Tessari I. Bubacco L. Dell’Acqua S. Casella L. (2013). Copper(I)-α-Synuclein Interaction: Structural Description of Two Independent and Competing Metal Binding Sites. Inorg. Chem. 52 (3), 1358–1367. 10.1021/ic302050m 23343468

[B24] Canerina-Amaro A. Pereda D. Diaz M. Rodriguez-Barreto D. Casañas-Sánchez V. Heffer M. (2019). Differential Aggregation and Phosphorylation of Alpha Synuclein in Membrane Compartments Associated with Parkinson Disease. Front. Neurosci. 13 (382), 1–21. 10.3389/fnins.2019.00382 31068782 PMC6491821

[B25] Cartelli D. Aliverti A. Barbiroli A. Santambrogio C. Ragg E. M. Casagrande F. V. M. (2016). α-Synuclein Is a Novel Microtubule Dynamase. Sci. Rep. 6 (33289), 1–13. 10.1038/srep33289 27628239 PMC5024109

[B26] Caruana M. Högen T. Levin J. Hillmer A. Giese A. Vassallo N. (2011). Inhibition and Disaggregation of α-synuclein Oligomers by Natural Polyphenolic Compounds. FEBS Lett. 585 (8), 1113–1120. 10.1016/j.febslet.2011.03.046 21443877

[B27] Chaari A. Eliezer D. Ladjimi M. (2016). The C-Terminal α-helices of Mammalian Hsc70 Play a Critical Role in the Stabilization of α-synuclein Binding and Inhibition of Aggregation. Int. J. Biol. Macromolecules 83, 433–441. 10.1016/j.ijbiomac.2015.10.089 PMC4876635 26601760

[B28] Chandra S. Chen X. Rizo J. Jahn R. Südhof T. C. (2003). A Broken α-Helix in Folded α-Synuclein. J. Biol. Chem. 278 (17), 15313–15318. 10.1074/jbc.M213128200 12586824

[B29] Chi Y.-C. Armstrong G. S. Jones D. N. M. Eisenmesser E. Z. Liu C.-W. (2014). Residue Histidine 50 Plays a Key Role in Protecting α-Synuclein from Aggregation at Physiological pH. J. Biol. Chem. 289 (22), 15474–15481. 10.1074/jbc.M113.544049 24742669 PMC4140903

[B30] Chidananda A. H. Sharma A. K. Khandelwal R. Sharma Y. (2019). Secretagogin Binding Prevents α-Synuclein Fibrillation. Biochemistry 58 (46), 4585–4589. 10.1021/acs.biochem.9b00656 31617346

[B31] Chiricozzi E. Lunghi G. Di Biase E. Fazzari M. Sonnino S. Mauri L. (2020). GM1 Ganglioside Is a Key Factor in Maintaining the Mammalian Neuronal Functions Avoiding Neurodegeneration. Ijms 21 (3), 868–929. 10.3390/ijms21030868 PMC7037093 32013258

[B32] Ciechanover A. Kwon Y. T. (2015). Degradation of Misfolded Proteins in Neurodegenerative Diseases: Therapeutic Targets and Strategies. Exp. Mol. Med. 47, e147. 10.1038/emm.2014.117 25766616 PMC4351408

[B33] Conway K. A. Harper J. D. Lansbury P. T. (1998). Accelerated *In Vitro* Fibril Formation by a Mutant α-synuclein Linked to Early-Onset Parkinson Disease. Nat. Med. 4 (11), 1318–1320. 10.1038/3311 9809558

[B34] Coskuner O. Wise-Scira O. (2013). Structures and Free Energy Landscapes of the A53T Mutant-type α-Synuclein Protein and Impact of A53T Mutation on the Structures of the Wild-type α-Synuclein Protein with Dynamics. ACS Chem. Neurosci. 4 (7), 1101–1113. 10.1021/cn400041j 23607785 PMC3715894

[B35] Daniels M. J. Nourse J. B. Kim H. Sainati V. Schiavina M. Murrali M. G. (2019). Cyclized NDGA Modifies Dynamic α-synuclein Monomers Preventing Aggregation and Toxicity. Sci. Rep. 9 (1), 1–17. 10.1038/s41598-019-39480-z 30814575 PMC6393491

[B36] Davidson W. S. Jonas A. Clayton D. F. George J. M. (1998). Stabilization of α-Synuclein Secondary Structure upon Binding to Synthetic Membranes. J. Biol. Chem. 273 (16), 9443–9449. 10.1074/jbc.273.16.9443 9545270

[B37] De Ricco R. Valensin D. Dell’Acqua S. Casella L. Hureau C. Faller P. (2015). Copper(I/II), α/β-Synuclein and Amyloid-β: Menage à Trois? ChemBioChem 16 (16), 2319–2328. 10.1002/cbic.201500425 26338312

[B38] Dedmon M. M. Christodoulou J. Wilson M. R. Dobson C. M. (2005a). Heat Shock Protein 70 Inhibits α-Synuclein Fibril Formation via Preferential Binding to Prefibrillar Species. J. Biol. Chem. 280 (15), 14733–14740. 10.1074/jbc.M413024200 15671022

[B39] Dedmon M. M. Lindorff-Larsen K. Christodoulou J. Vendruscolo M. Dobson C. M. (2005b). Mapping Long-Range Interactions in α-Synuclein Using Spin-Label NMR and Ensemble Molecular Dynamics Simulations. J. Am. Chem. Soc. 127 (2), 476–477. 10.1021/ja044834j 15643843

[B40] Dučić T. Carboni E. Lai B. Chen S. Michalke B. Lázaro D. F. (2015). Alpha-Synuclein Regulates Neuronal Levels of Manganese and Calcium. ACS Chem. Neurosci. 6 (10), 1769–1779. 10.1021/acschemneuro.5b00093 26284970

[B41] Dułak D. Gadzała M. Banach M. Konieczny L. Roterman I. (2020). Alternative Structures of α-Synuclein. Molecules 25 (3). 10.3390/molecules25030600 PMC7038196 32019169

[B42] Ebrahimi-Fakhari D. Wahlster L. McLean P. J. (2011). Molecular Chaperones in Parkinson’s Disease—Present and Future. J. Parkinson’s Dis. 1 (4), 299–320. 10.3233/JPD-2011-11044 22279517 PMC3264060

[B43] Eliezer D. Kutluay E. Bussell R. Browne G. (2001). Conformational Properties of α-synuclein in its Free and Lipid-Associated States 1 1Edited by P. E. Wright. J. Mol. Biol. 307 (4), 1061–1073. 10.1006/jmbi.2001.4538 11286556

[B44] Eliezer D. (2009). Biophysical Characterization of Intrinsically Disordered Proteins. Curr. Opin. Struct. Biol. 19 (1), 23–30. 10.1016/j.sbi.2008.12.004 19162471 PMC2728036

[B45] Fakhree M. A. A. Nolten I. S. Blum C. Claessens M. M. A. E. (2018). Different Conformational Subensembles of the Intrinsically Disordered Protein α-Synuclein in Cells. J. Phys. Chem. Lett. 9 (6), 1249–1253. 10.1021/acs.jpclett.8b00092 29474083 PMC5857923

[B46] Fantini J. Carlus D. Yahi N. (2011). The Fusogenic Tilted Peptide (67-78) of α-synuclein Is a Cholesterol Binding Domain. Biochim. Biophys. Acta (Bba) - Biomembranes 1808 (10), 2343–2351. 10.1016/j.bbamem.2011.06.017 21756873

[B47] Fares M.-B. Ait-Bouziad N. Dikiy I. Mbefo M. K. Jovii A. Kiely A. (2014). The Novel Parkinson's Disease Linked Mutation G51D Attenuates *In Vitro* Aggregation and Membrane Binding of -synuclein, and Enhances its Secretion and Nuclear Localization in Cells. Hum. Mol. Genet. 23 (17), 4491–4509. 10.1093/hmg/ddu165 24728187 PMC4119404

[B48] Fauvet B. Mbefo M. K. Fares M.-B. Desobry C. Michael S. Ardah M. T. (2012). α-Synuclein in Central Nervous System and from Erythrocytes, Mammalian Cells, and *Escherichia coli* Exists Predominantly as Disordered Monomer*. J. Biol. Chem. 287 (19), 15345–15364. 10.1074/jbc.M111.318949 22315227 PMC3346117

[B49] Fields C. R. Bengoa-Vergniory N. Wade-Martins R. (2019). Targeting Alpha-Synuclein as a Therapy for Parkinson's Disease. Front. Mol. Neurosci. 12, 1–14. 10.3389/fnmol.2019.00299 31866823 PMC6906193

[B50] Fortin D. L. Troyer M. D. Nakamura K. Kubo S. I. Anthony M. D. Edwards R. H. (2004). Lipid Rafts Mediate the Synaptic Localization of -Synuclein. J. Neurosci. 24 (30), 6715–6723. 10.1523/JNEUROSCI.1594-04.2004 15282274 PMC6729723

[B51] Fredenburg R. A. Rospigliosi C. Meray R. K. Kessler J. C. Lashuel H. A. Eliezer D. (2007). The Impact of the E46K Mutation on the Properties of α-Synuclein in its Monomeric and Oligomeric States. Biochemistry 46 (24), 7107–7118. 10.1021/bi7000246 17530780

[B52] Fusco G. De Simone A. Gopinath T. Vostrikov V. Vendruscolo M. Dobson C. M. (2014). Direct Observation of the Three Regions in α-synuclein that Determine its Membrane-Bound Behaviour. Nat. Commun. 5 (3827), 1–8. 10.1038/ncomms4827 PMC4046108 24871041

[B53] Galvagnion C. Buell A. K. Meisl G. Michaels T. C. T. Vendruscolo M. Knowles T. P. J. (2015). Lipid Vesicles Trigger α-synuclein Aggregation by Stimulating Primary Nucleation. Nat. Chem. Biol. 11 (3), 229–234. 10.1038/nchembio.1750 25643172 PMC5019199

[B54] Galvagnion C. Brown J. W. P. Ouberai M. M. Flagmeier P. Vendruscolo M. Buell A. K. (2016). Chemical Properties of Lipids Strongly Affect the Kinetics of the Membrane-Induced Aggregation of α-synuclein. Proc. Natl. Acad. Sci. USA 113 (26), 7065–7070. 10.1073/pnas.1601899113 27298346 PMC4932957

[B55] Gao X. Carroni M. Nussbaum-Krammer C. Mogk A. Nillegoda N. B. Szlachcic A. (2015). Human Hsp70 Disaggregase Reverses Parkinson's-Linked α-Synuclein Amyloid Fibrils. Mol. Cel 59 (5), 781–793. 10.1016/j.molcel.2015.07.012 PMC5072489 26300264

[B56] Garza-Lombó C. Posadas Y. Quintanar L. Gonsebatt M. E. Franco R. (2018). Neurotoxicity Linked to Dysfunctional Metal Ion Homeostasis and Xenobiotic Metal Exposure: Redox Signaling and Oxidative Stress. Antioxid. Redox Signal. 28 (18), 1669–1703. 10.1089/ars.2017.7272 29402131 PMC5962337

[B57] Ghio S. Camilleri A. Caruana M. Ruf V. C. Schmidt F. Leonov A. (2019). Cardiolipin Promotes Pore-Forming Activity of Alpha-Synuclein Oligomers in Mitochondrial Membranes. ACS Chem. Neurosci. 10 (8), 3815–3829. 10.1021/acschemneuro.9b00320 31356747

[B58] Ghosh D. Mondal M. Mohite G. M. Singh P. K. Ranjan P. Anoop A. (2013). The Parkinson's Disease-Associated H50Q Mutation Accelerates α-Synuclein Aggregation *In Vitro* . Biochemistry 52 (40), 6925–6927. 10.1021/bi400999d 24047453

[B59] Ghosh D. Sahay S. Ranjan P. Salot S. Mohite G. M. Singh P. K. (2014). The Newly Discovered Parkinson's Disease Associated Finnish Mutation (A53E) Attenuates α-Synuclein Aggregation and Membrane Binding. Biochemistry 53 (41), 6419–6421. 10.1021/bi5010365 25268550

[B60] Ghosh S. Kundu A. Chattopadhyay K. (2018). Small Molecules Attenuate the Interplay between Conformational Fluctuations, Early Oligomerization and Amyloidosis of Alpha Synuclein. Sci. Rep. 8 (1), 1–16. 10.1038/s41598-018-23718-3 29615762 PMC5882917

[B61] Grassi S. Giussani P. Mauri L. Prioni S. Sonnino S. Prinetti A. (2020). Lipid Rafts and Neurodegeneration: Structural and Functional Roles in Physiologic Aging and Neurodegenerative Diseases. J. Lipid Res. 61 (5), 636–654. 10.1194/jlr.TR119000427 31871065 PMC7193971

[B62] Guerrero-Ferreira R. Taylor N. M. Mona D. Ringler P. Lauer M. E. Riek R. (2018). Cryo-EM Structure of Alpha-Synuclein Fibrils. ELife 7, e36402. 10.7554/eLife.36402 29969391 PMC6092118

[B63] Han J. Y. Choi T. S. Kim H. I. (2018). Molecular Role of Ca2+ and Hard Divalent Metal Cations on Accelerated Fibrillation and Interfibrillar Aggregation of α-Synuclein. Sci. Rep. 8 (1), 1–11. 10.1038/s41598-018-20320-5 29382893 PMC5789889

[B64] Heise H. Hoyer W. Becker S. Andronesi O. C. Riedel D. Baldus M. (2005). Molecular-level Secondary Structure, Polymorphism, and Dynamics of Full-Length -synuclein Fibrils Studied by Solid-State NMR. Proc. Natl. Acad. Sci. 102 (44), 15871–15876. 10.1073/pnas.0506109102 16247008 PMC1276071

[B65] Högen T. Levin J. Schmidt F. Caruana M. Vassallo N. Kretzschmar H. (2012). Two Different Binding Modes of α-Synuclein to Lipid Vesicles Depending on its Aggregation State. Biophysical J. 102 (7), 1646–1655. 10.1016/j.bpj.2012.01.059 PMC3318129 22500765

[B66] Ibrahim T. McLaurin J. (2016). α-Synuclein Aggregation, Seeding and Inhibition by Scyllo-Inositol. Biochem. Biophysical Res. Commun. 469 (3), 529–534. 10.1016/j.bbrc.2015.12.043 26697752

[B67] Ilie I. M. Caflisch A. (2019). Simulation Studies of Amyloidogenic Polypeptides and Their Aggregates. Chem. Rev. 119 (12), 6956–6993. 10.1021/acs.chemrev.8b00731 30973229

[B68] Iyer A. Roeters S. J. Kogan V. Woutersen S. Claessens M. M. A. E. Subramaniam V. (2017). C-terminal Truncated α-Synuclein Fibrils Contain Strongly Twisted β-Sheets. J. Am. Chem. Soc. 139 (43), 15392–15400. 10.1021/jacs.7b07403 28968082 PMC5668890

[B69] Jakubec M. Bariås E. Furse S. Govasli M. L. George V. Turcu D. (2019). Cholesterol Is a strong Promotor of an α-Synuclein Membrane Binding Mode that Accelerates Oligomerization. BioRxiv 12, 725762. 10.1101/725762

[B70] Jao C. C. Hegde B. G. Chen J. Haworth I. S. Langen R. (2008). Structure of Membrane-Bound -synuclein from Site-Directed Spin Labeling and Computational Refinement. Proc. Natl. Acad. Sci. 105 (50), 19666–19671. 10.1073/pnas.0807826105 19066219 PMC2605001

[B71] Javed H. Ojha S. (2020). Therapeutic Potential of Baicalein in Parkinson's Disease: Focus on Inhibition of α-Synuclein Oligomerization and Aggregation. Synucleins - Biochem. Role Dis. 14, 1–16. 10.5772/intechopen.83589

[B72] Ji K. Zhao Y. Yu T. Wang Z. Gong H. Yang X. (2016). Inhibition Effects of Tanshinone on the Aggregation of α-synuclein. Food Funct. 7 (1), 409–416. 10.1039/c5fo00664c 26456030

[B73] Jónsson S. A. Mohanty S. Irbäck A. (2012). Distinct Phases of Free α-synuclein-A Monte Carlo Study. Proteins 80 (9), 2169–2177. 10.1002/prot.24107 22552968

[B74] Kardani J. Roy I. (2015). Understanding Caffeine's Role in Attenuating the Toxicity of α-Synuclein Aggregates: Implications for Risk of Parkinson's Disease. ACS Chem. Neurosci. 6 (9), 1613–1625. 10.1021/acschemneuro.5b00158 26167732

[B75] Kardani J. Sethi R. Roy I. (2017). Nicotine Slows Down Oligomerisation of α-synuclein and Ameliorates Cytotoxicity in a Yeast Model of Parkinson's Disease. Biochim. Biophys. Acta (Bba) - Mol. Basis Dis. 1863 (6), 1454–1463. 10.1016/j.bbadis.2017.02.002 28167231

[B76] Kauzmann W. (1954). “Denaturation of Proteins and Enzymes,” in The Mechanism of Enzyme Action, Berlin: Springer, 71.

[B77] Khalaf O. Fauvet B. Oueslati A. Dikiy I. Mahul-Mellier A.-L. Ruggeri F. S. (2014). The H50Q Mutation Enhances α-Synuclein Aggregation, Secretion, and Toxicity. J. Biol. Chem. 289 (32), 21856–21876. 10.1074/jbc.M114.553297 24936070 PMC4139205

[B78] Kiechle M. Grozdanov V. Danzer K. M. (2020). The Role of Lipids in the Initiation of α-Synuclein Misfolding. Front. Cel Dev. Biol. 8, 957. 10.3389/fcell.2020.562241 PMC7523214 33042996

[B79] Kiely A. P. Asi Y. T. Kara E. Limousin P. Ling H. Lewis P. (2013). α-Synucleinopathy Associated with G51D SNCA Mutation: a Link between Parkinson's Disease and Multiple System Atrophy? Acta Neuropathol. 125 (5), 753–769. 10.1007/s00401-013-1096-7 23404372 PMC3681325

[B80] Kim D.-H. Lee J. Mok K. Lee J. Han K.-H. (2020). Salient Features of Monomeric Alpha-Synuclein Revealed by NMR Spectroscopy. Biomolecules 10 (3), 428–515. 10.3390/biom10030428 PMC7175124 32164323

[B81] Klucken J. Outeiro T. F. Nguyen P. McLean P. J. Hyman B. T. (2006). Detection of Novel Intracellular O‐synuclein Oligomeric Species by Fluorescence Lifetime Imaging. FASEB j. 20 (12), 2050–2057. 10.1096/fj.05-5422com 17012257

[B82] Kostka M. Högen T. Danzer K. M. Levin J. Habeck M. Wirth A. (2008). Single Particle Characterization of Iron-Induced Pore-Forming α-Synuclein Oligomers. J. Biol. Chem. 283 (16), 10992–11003. 10.1074/jbc.M709634200 18258594

[B83] Krüger R. Kuhn W. Müller T. Woitalla D. Graeber M. Kosel S. (1998). Ala30Pro Mutation in the Gene Encoding A-Synuclein in Parkinson’s Disease. Nat. Genet. 18 (3), 231–236. 10.1038/ng0298-106 9462735

[B84] Kumar S. Sarkar A. Sundar D. (2009). Controlling Aggregation Propensity in A53T Mutant of Alpha-Synuclein Causing Parkinson's Disease. Biochem. Biophys. Res. Commun. 387 (2), 305–309. 10.1016/j.bbrc.2009.07.008 19580781

[B85] Kurnik M. Sahin C. Andersen C. B. Lorenzen N. Giehm L. Mohammad-Beigi H. (2018). Potent α-Synuclein Aggregation Inhibitors, Identified by High-Throughput Screening, Mainly Target the Monomeric State. Cel Chem. Biol. 25 (11), 1389–1402. 10.1016/j.chembiol.2018.08.005 30197194

[B86] Lamberto G. R. Binolfi A. Orcellet M. L. Bertoncini C. W. Zweckstetter M. Griesinger C. (2009). Structural and Mechanistic Basis behind the Inhibitory Interaction of PcTS on α-synuclein Amyloid Fibril Formation. Pnas 106 (50), 21057–21062. 10.1073/pnas.0902603106 19948969 PMC2795524

[B87] Lautenschläger J. Stephens A. D. Fusco G. Ströhl F. Curry N. Zacharopoulou M. (2018). C-terminal Calcium Binding of α-synuclein Modulates Synaptic Vesicle Interaction. Nat. Commun. 9 (1), 712. 10.1038/s41467-018-03111-4 29459792 PMC5818535

[B88] Lesage S. Anheim M. Letournel F. Bousset L. Honoré A. Rozas N. (2013). G51D α-synuclein Mutation Causes a Novel Parkinsonian-Pyramidal Syndrome. Ann. Neurol. 73 (4), 459–471. 10.1002/ana.23894 23526723

[B89] Lorentzon E. Kumar R. Horvath I. Wittung-Stafshede P. (2020). Differential Effects of Cu2+ and Fe3+ Ions on *In Vitro* Amyloid Formation of Biologically-Relevant α-synuclein Variants. Biometals 33, 97–106. 10.1007/s10534-020-00234-4 32170541 PMC7295844

[B90] Lorenzen N. Lemminger L. Pedersen J. N. Nielsen S. B. Otzen D. E. (2014). The N-Terminus of α-synuclein Is Essential for Both Monomeric and Oligomeric Interactions with Membranes. FEBS Lett. 588 (3), 497–502. 10.1016/j.febslet.2013.12.015 24374342

[B91] Lothian A. Lago L. Mukherjee S. Connor A. R. Fowler C. McLean C. A. (2019). Characterization of the Metal Status of Natively Purified Alpha-Synuclein from Human Blood, Brain Tissue, or Recombinant Sources Using Size Exclusion ICP-MS Reveals No Significant Binding of Cu, Fe or Zn. Metallomics 11 (1), 128–140. 10.1039/c8mt00223a 30465671

[B92] Lu Y. Prudent M. Fauvet B. Lashuel H. A. Girault H. H. (2011). Phosphorylation of α-Synuclein at Y125 and S129 Alters its Metal Binding Properties: Implications for Understanding the Role of α-Synuclein in the Pathogenesis of Parkinson's Disease and Related Disorders. ACS Chem. Neurosci. 2 (11), 667–675. 10.1021/cn200074d 22860160 PMC3369716

[B93] Luk K. C. Mills I. P. Trojanowski J. Q. Lee V. M.-Y. (2008). Interactions between Hsp70 and the Hydrophobic Core of α-Synuclein Inhibit Fibril Assembly. Biochemistry 47 (47), 12614–12625. 10.1021/bi801475r 18975920 PMC2648307

[B94] Ma L. Yang C. Zheng J. Chen Y. Xiao Y. Huang K. (2020). Non-polyphenolic Natural Inhibitors of Amyloid Aggregation. Eur. J. Med. Chem. 192, 112197. 10.1016/j.ejmech.2020.112197 32172082

[B95] Månsson C. Arosio P. Hussein R. Kampinga H. H. Hashem R. M. Boelens W. C. (2014). Interaction of the Molecular Chaperone DNAJB6 with Growing Amyloid-Beta 42 (Aβ42) Aggregates Leads to Sub-stoichiometric Inhibition of Amyloid Formation. J. Biol. Chem. 289 (45), 31066–31076. 10.1074/jbc.M114.595124 25217638 PMC4223311

[B96] Man W. K. De Simone A. Barritt J. D. Vendruscolo M. Dobson C. M. Fusco G. (2020). A Role of Cholesterol in Modulating the Binding of α-Synuclein to Synaptic-like Vesicles. Front. Neurosci. 14, 1–11. 10.3389/fnins.2020.00018 32063829 PMC7000551

[B97] Martial B. Lefèvre T. Buffeteau T. Auger M. (2019). Vibrational Circular Dichroism Reveals Supramolecular Chirality Inversion of α-Synuclein Peptide Assemblies upon Interactions with Anionic Membranes. ACS Nano 13 (3), 3232–3242. 10.1021/acsnano.8b08932 30811930

[B98] Martial B. Raîche-Marcoux G. Lefèvre T. Audet P. Voyer N. Auger M. (2020). Structure of a Parkinson's Disease-Involved α-Synuclein Peptide Is Modulated by Membrane Composition and Physical State. J. Phys. Chem. B. 124 (17), 3469–3481. 10.1021/acs.jpcb.0c00945 32227952

[B99] Martinez Z. Zhu M. Han S. Fink A. L. (2007). GM1 Specifically Interacts with α-Synuclein and Inhibits Fibrillation. Biochemistry 46 (7), 1868–1877. 10.1021/bi061749a 17253773

[B100] Mazzulli J. R. Armakola M. Dumoulin M. Parastatidis I. Ischiropoulos H. (2007). Cellular Oligomerization of α-Synuclein Is Determined by the Interaction of Oxidized Catechols with a C-Terminal Sequence. J. Biol. Chem. 282 (43), 31621–31630. 10.1074/jbc.M704737200 17785456

[B101] McDowall J. S. Brown D. R. (2016). Alpha-synuclein: Relating Metals to Structure, Function and Inhibition. Metallomics 8 (4), 385–397. 10.1039/c6mt00026f 26864076

[B102] McLean P. J. Kawamata H. Ribich S. Hyman B. T. (2000). Membrane Association and Protein Conformation of α-Synuclein in Intact Neurons. J. Biol. Chem. 275 (12), 8812–8816. 10.1074/jbc.275.12.8812 10722726

[B103] Meade R. M. Fairlie D. P. Mason J. M. (2019). Alpha-synuclein Structure and Parkinson's Disease - Lessons and Emerging Principles. Mol. Neurodegener. 14 (1), 29. 10.1186/s13024-019-0329-1 31331359 PMC6647174

[B104] Mesa-Herrera F. Taoro-González L. Valdés-Baizabal C. Diaz M. Marín R. (2019). Lipid and Lipid Raft Alteration in Aging and Neurodegenerative Diseases: A Window for the Development of New Biomarkers. Ijms 20 (15), 3810. 10.3390/ijms20153810 PMC6696273 31382686

[B105] Miotto M. C. Binolfi A. Zweckstetter M. Griesinger C. Fernández C. O. (2014). Bioinorganic Chemistry of Synucleinopathies: Deciphering the Binding Features of Met Motifs and His-50 in AS-Cu(I) Interactions. J. Inorg. Biochem. 141, 208–211. 10.1016/j.jinorgbio.2014.08.012 25218565

[B106] Mirecka E. A. Shaykhalishahi H. Gauhar A. Akgül Ş. Lecher J. Willbold D. (2014). Sequestration of a β-Hairpin for Control of α-Synuclein Aggregation. Angew. Chem. Int. Ed. 53 (16), 4227–4230. 10.1002/anie.201309001 24623599

[B107] Mori A. Imai Y. Hattori N. (2020). Lipids: Key Players that Modulate α-Synuclein Toxicity and Neurodegeneration in Parkinson's Disease. Ijms 21 (9), 3301. 10.3390/ijms21093301 PMC7247581 32392751

[B108] Morshedi D. Aliakbari F. Tayaranian-Marvian A. Fassihi A. Pan-Montojo F. Pérez-Sánchez H. (2015). Cuminaldehyde as the Major Component ofCuminum Cyminum, a Natural Aldehyde with Inhibitory Effect on Alpha-Synuclein Fibrillation and Cytotoxicity. J. Food Sci. 80 (10), H2336–H2345. 10.1111/1750-3841.13016 26351865

[B109] Narhi L. Wood S. J. Steavenson S. Jiang Y. Wu G. M. Anafi D. (1999). Both Familial Parkinson's Disease Mutations Accelerate α-Synuclein Aggregation. J. Biol. Chem. 274 (14), 9843–9846. 10.1074/jbc.274.14.9843 10092675

[B110] Nath A. Sammalkorpi M. Dewitt D. C. Trexler A. J. Elbaum-Garfinkle S. O’Hern C. S. (2012). The Conformational Ensembles of α-Synuclein and Tau: Combining Single-Molecule FRET and Simulations. Biophysical J. 103 (9), 1940–1949. 10.1016/j.bpj.2012.09.032 PMC3491709 23199922

[B111] O’Leary E. I. Lee J. C. (2019). Interplay between α-synuclein Amyloid Formation and Membrane Structure. Biochim. Biophys. Acta - Proteins Proteomics 1867 (5), 483–491. 10.1016/j.bbapap.2018.09.012 30287222 PMC6445794

[B112] Okita Y. Rcom-H'cheo-Gauthier A. N. Goulding M. Chung R. S. Faller P. Pountney D. L. (2017). Metallothionein, Copper and Alpha-Synuclein in Alpha-Synucleinopathies. Front. Neurosci. 11, 114. 10.3389/fnins.2017.00114 28420950 PMC5380005

[B113] Okuwaki R. Shinmura I. Morita S. Matsugami A. Hayashi F. Goto Y. (2020). Distinct Residual and Disordered Structures of Alpha-Synuclein Analyzed by Amide-Proton Exchange and NMR Signal Intensity. Biochim. Biophys. Acta (Bba) - Proteins Proteomics 1868 (9), 140464. 10.1016/j.bbapap.2020.140464 32497661

[B114] Oliveri V. (2019). Toward the Discovery and Development of Effective Modulators of α-synuclein Amyloid Aggregation. Eur. J. Med. Chem. 167, 10–36. 10.1016/j.ejmech.2019.01.045 30743095

[B115] Ouberai M. M. Wang J. Swann M. J. Galvagnion C. Guilliams T. Dobson C. M. (2013). α-Synuclein Senses Lipid Packing Defects and Induces Lateral Expansion of Lipids Leading to Membrane Remodeling. J. Biol. Chem. 288 (29), 20883–20895. 10.1074/jbc.M113.478297 23740253 PMC3774359

[B116] Pasanen P. Myllykangas L. Siitonen M. Raunio A. Kaakkola S. Lyytinen J. (2014). A Novel α-synuclein Mutation A53E Associated with Atypical Multiple System Atrophy and Parkinson's Disease-type Pathology. Neurobiol. Aging 35 (9), 2180.e1–2180.e5. 10.1016/j.neurobiolaging.2014.03.024 24746362

[B117] Paul A. Zhang B.-D. Mohapatra S. Li G. Li Y.-M. Gazit E. (2019). Novel Mannitol-Based Small Molecules for Inhibiting Aggregation of α-Synuclein Amyloids in Parkinson's Disease. Front. Mol. Biosci. 6, 1–10. 10.3389/fmolb.2019.00016 30968030 PMC6438916

[B118] Perni M. Galvagnion C. Maltsev A. Meisl G. Müller M. B. D. Challa P. K. (2017). A Natural Product Inhibits the Initiation of α-synuclein Aggregation and Suppresses its Toxicity. Proc. Natl. Acad. Sci. USA 114 (6), E1009–E1017. 10.1073/pnas.1610586114 28096355 PMC5307473

[B119] Pfrieger F. W. (2003). Role of Cholesterol in Synapse Formation and Function. Biochim. Biophys. Acta (Bba) - Biomembr. 1610 (2), 271–280. 10.1016/S0005-2736(03)00024-5 12648780

[B120] Pineda A. Burré J. (2017). Modulating Membrane Binding of α-synuclein as a Therapeutic Strategy. Proc. Natl. Acad. Sci. USA 114 (6), 1223–1225. 10.1073/pnas.1620159114 28126719 PMC5307480

[B121] Pirc K. Ulrih N. P. (2015). α-Synuclein Interactions with Phospholipid Model Membranes: Key Roles for Electrostatic Interactions and Lipid-Bilayer Structure. Biochim. Biophys. Acta (Bba) - Biomembr. 1848 (10), 2002–2012. 10.1016/j.bbamem.2015.06.021 26119565

[B122] Plotegher N. Greggio E. Bisaglia M. Bubacco L. (2014). Biophysical Groundwork as a Hinge to Unravel the Biology of α-synuclein Aggregation and Toxicity. Quart. Rev. Biophys. 47 (1), 1–48. 10.1017/S0033583513000097 24443929

[B123] Polymeropoulos M. H. Lavedan C. Leroy E. Ide S. E. Dehejia A. Dutra A. (1997). Mutation in the -Synuclein Gene Identified in Families with Parkinson's Disease. Science 276 (5321), 2045–2047. 10.1126/science.276.5321.2045 9197268

[B124] Post M. R. Lieberman O. J. Mosharov E. V. (2018). Can Interactions between α-Synuclein, Dopamine and Calcium Explain Selective Neurodegeneration in Parkinson's Disease? Front. Neurosci. 12, 161. 10.3389/fnins.2018.00161 29593491 PMC5861202

[B125] Pujols J. Peña-Díaz S. Lázaro D. F. Peccati F. Pinheiro F. González D. (2018). Small Molecule Inhibits α-synuclein Aggregation, Disrupts Amyloid Fibrils, and Prevents Degeneration of Dopaminergic Neurons. Proc. Natl. Acad. Sci. USA 115 (41), 10481–10486. 10.1073/pnas.1804198115 30249646 PMC6187188

[B126] Ramis R. Ortega-Castro J. Vilanova B. Adrover M. Frau J. (2017). Copper(II) Binding Sites in N-Terminally Acetylated α-Synuclein: A Theoretical Rationalization. J. Phys. Chem. A. 121 (30), 5711–5719. 10.1021/acs.jpca.7b03165 28691818

[B127] Rcom-H’cheo-Gauthier A. Goodwin J. Pountney D. L. (2014). Interactions between Calcium and Alpha-Synuclein in Neurodegeneration. Biomolecules 4 (3), 795–811. 10.3390/biom4030795 25256602 PMC4192672

[B128] Rekas A. Knott R. B. Sokolova A. Barnham K. J. Perez K. A. Masters C. L. (2010). The Structure of Dopamine Induced α-synuclein Oligomers. Eur. Biophys. J. 39 (10), 1407–1419. 10.1007/s00249-010-0595-x 20309679

[B129] Ren B. Liu Y. Zhang Y. Zhang M. Sun Y. Liang G. (2017). Tanshinones Inhibit hIAPP Aggregation, Disaggregate Preformed hIAPP Fibrils, and Protect Cultured Cells. J. Mater. Chem. B. 6 (1), 56–67. 10.1039/c7tb02538f 32254193

[B130] Robotta M. Braun P. Van Rooijen B. Subramaniam V. Huber M. Drescher M. (2011). Direct Evidence of Coexisting Horseshoe and Extended helix Conformations of Membrane-Bound Alpha-Synuclein. ChemPhysChem 12 (2), 267–269. 10.1002/cphc.201000815 21275016

[B131] Robotta M. Cattani J. Martins J. C. Subramaniam V. Drescher M. (2017). Alpha-Synuclein Disease Mutations Are Structurally Defective and Locally Affect Membrane Binding. J. Am. Chem. Soc. 139 (12), 4254–4257. 10.1021/jacs.6b05335 28298083

[B132] Rospigliosi C. C. McClendon S. Schmid A. W. Ramlall T. F. Barré P. Lashuel H. A. (2009). E46K Parkinson's-Linked Mutation Enhances C-Terminal-To-N-Terminal Contacts in α-Synuclein. J. Mol. Biol. 388 (5), 1022–1032. 10.1016/j.jmb.2009.03.065 19345692 PMC2719283

[B133] Runfola M. De Simone A. Vendruscolo M. Dobson C. M. Fusco G. (2020). The N-Terminal Acetylation of α-Synuclein Changes the Affinity for Lipid Membranes but Not the Structural Properties of the Bound State. Sci. Rep. 10 (1), 1–10. 10.1038/s41598-019-57023-4 31937832 PMC6959233

[B134] Ryan T. Bamm V. V. Stykel M. G. Coackley C. L. Humphries K. M. Jamieson-Williams R. (2018). Cardiolipin Exposure on the Outer Mitochondrial Membrane Modulates α-synuclein. Nat. Commun. 9 (1), 1–17. 10.1038/s41467-018-03241-9 29483518 PMC5827019

[B135] Schneider J. S. Aras R. Williams C. K. Koprich J. B. Brotchie J. M. Singh V. (2019). GM1 Ganglioside Modifies α-Synuclein Toxicity and Is Neuroprotective in a Rat α-Synuclein Model of Parkinson's Disease. Sci. Rep. 9 (1), 1–12. 10.1038/s41598-019-42847-x 31182727 PMC6557812

[B136] Sebastião A. M. Colino-Oliveira M. Assaife-Lopes N. Dias R. B. Ribeiro J. A. (2013). Lipid Rafts, Synaptic Transmission and Plasticity: Impact in Age-Related Neurodegenerative Diseases. Neuropharmacology 64, 97–107. 10.1016/j.neuropharm.2012.06.053 22820274

[B137] Serpell L. C. Berriman J. Jakes R. Goedert M. Crowther R. A. (2000). Fiber Diffraction of Synthetic Alpha -synuclein Filaments Shows Amyloid-like Cross-Beta Conformation. Proc. Natl. Acad. Sci. 97 (9), 4897–4902. 10.1073/pnas.97.9.4897 10781096 PMC18329

[B138] Sezgin E. Levental I. Mayor S. Eggeling C. (2017). The Mystery of Membrane Organization: Composition, Regulation and Roles of Lipid Rafts. Nat. Rev. Mol. Cel Biol. 18 (6), 361–374. 10.1038/nrm.2017.16 PMC5500228 28356571

[B139] Shaltiel-Karyo R. Frenkel-Pinter M. Rockenstein E. Patrick C. Levy-Sakin M. Schiller A. (2013). A Blood-Brain Barrier (BBB) Disrupter Is Also a Potent α-Synuclein (α-Syn) Aggregation Inhibitor. J. Biol. Chem. 288 (24), 17579–17588. 10.1074/jbc.M112.434787 23637226 PMC3682557

[B140] Shvadchak V. V. Falomir-Lockhart L. J. Yushchenko D. A. Jovin T. M. (2011). Specificity and Kinetics of α-Synuclein Binding to Model Membranes Determined with Fluorescent Excited State Intramolecular Proton Transfer (ESIPT) Probe. J. Biol. Chem. 286 (15), 13023–13032. 10.1074/jbc.M110.204776 21330368 PMC3075648

[B141] Shvadchak V. V. Afitska K. Yushchenko D. A. (2018). Inhibition of α-Synuclein Amyloid Fibril Elongation by Blocking Fibril Ends. Angew. Chem. Int. Ed. 57 (20), 5690–5694. 10.1002/anie.201801071 29575453

[B142] Stephens A. D. Zacharopoulou M. Kaminski Schierle G. S. (2019). The Cellular Environment Affects Monomeric α-Synuclein Structure. Trends Biochem. Sci. 44 (5), 453–466. 10.1016/j.tibs.2018.11.005 30527975

[B143] Sternke-Hoffmann R. Peduzzo A. Bolakhrif N. Haas R. Buell A. K. (2020). The Aggregation Conditions Define whether EGCG Is an Inhibitor or Enhancer of α-Synuclein Amyloid Fibril Formation. Ijms 21 (6), 1995. 10.3390/ijms21061995 PMC7139648 32183378

[B144] Stöckl M. Fischer P. Wanker E. Herrmann A. (2008). α-Synuclein Selectively Binds to Anionic Phospholipids Embedded in Liquid-Disordered Domains. J. Mol. Biol. 375 (5), 1394–1404. 10.1016/j.jmb.2007.11.051 18082181

[B145] Sung Y.-H. Eliezer D. (2018). Structure and Dynamics of the Extended-helix State of Alpha-Synuclein: Intrinsic Lability of the Linker Region. Protein Sci. 27 (7), 1314–1324. 10.1002/pro.3426 29663556 PMC6032355

[B146] Terakawa M. S. Lee Y.-H. Kinoshita M. Lin Y. Sugiki T. Fukui N. (2018). Membrane-induced Initial Structure of α-synuclein Control its Amyloidogenesis on Model Membranes. Biochim. Biophys. Acta (Bba) - Biomembr. 1860 (3), 757–766. 10.1016/j.bbamem.2017.12.011 29273335

[B147] Tsigelny I. F. Sharikov Y. Wrasidlo W. Gonzalez T. Desplats P. A. Crews L. (2012). Role of α-synuclein Penetration into the Membrane in the Mechanisms of Oligomer Pore Formation. FEBS J. 279 (6), 1000–1013. 10.1111/j.1742-4658.2012.08489.x 22251432 PMC3925782

[B148] Tsigelny I. F. Sharikov Y. Kouznetsova V. L. Greenberg J. P. Wrasidlo W. Overk C. (2015). Molecular Determinants of α-Synuclein Mutants' Oligomerization and Membrane Interactions. ACS Chem. Neurosci. 6 (3), 403–416. 10.1021/cn500332w 25561023 PMC4944825

[B149] Tuttle M. D. Comellas G. Nieuwkoop A. J. Covell D. J. Berthold D. A. Kloepper K. D. (2016). Solid-state NMR Structure of a Pathogenic Fibril of Full-Length Human α-synuclein. Nat. Struct. Mol. Biol. 23 (5), 409–415. 10.1038/nsmb.3194 27018801 PMC5034296

[B150] Uversky V. Eliezer D. (2009). Biophysics of Parkinsons Disease: Structure and Aggregation of α- Synuclein. Cpps 10 (5), 483–499. 10.2174/138920309789351921 PMC3786709 19538146

[B151] Uversky V. N. Li J. Fink A. L. (2001). Evidence for a Partially Folded Intermediate in α-Synuclein Fibril Formation. J. Biol. Chem. 276 (14), 10737–10744. 10.1074/jbc.M010907200 11152691

[B152] Uversky V. N. (2013). The Most Important Thing Is the Tail: Multitudinous Functionalities of Intrinsically Disordered Protein Termini. FEBS Lett. 587 (13), 1891–1901. 10.1016/j.febslet.2013.04.042 23665034

[B153] Valiente-Gabioud A. A. Miotto M. C. Chesta M. E. Lombardo V. Binolfi A. Fernández C. O. (2016). Phthalocyanines as Molecular Scaffolds to Block Disease-Associated Protein Aggregation. Acc. Chem. Res. 49 (5), 801–808. 10.1021/acs.accounts.5b00507 27136297

[B154] Vasili E. Dominguez-Meijide A. Outeiro T. F. (2019). Spreading of α-Synuclein and Tau: A Systematic Comparison of the Mechanisms Involved. Front. Mol. Neurosci. 12, 1–23. 10.3389/fnmol.2019.00107 31105524 PMC6494944

[B155] Vilar M. Chou H.-T. Lührs T. Maji S. K. Riek-Loher D. Verel R. (2008). The Fold of -synuclein Fibrils. Proc. Natl. Acad. Sci. 105 (25), 8637–8642. 10.1073/pnas.0712179105 18550842 PMC2438424

[B156] Weinreb P. H. Zhen W. Poon A. W. Conway K. A. Lansbury P. T. (1996). NACP, A Protein Implicated in Alzheimer's Disease and Learning, Is Natively Unfolded. Biochemistry 35 (43), 13709–13715. 10.1021/bi961799n 8901511

[B157] Wise-Scira O. Aloglu A. K. Dunn A. Sakallioglu I. T. Coskuner O. (2013). Structures and Free Energy Landscapes of the Wild-type and A30P Mutant-type α-Synuclein Proteins with Dynamics. ACS Chem. Neurosci. 4 (3), 486–497. 10.1021/cn300198q 23374072 PMC3607329

[B158] Wu K.-P. Baum J. (2010). Detection of Transient Interchain Interactions in the Intrinsically Disordered Protein α-Synuclein by NMR Paramagnetic Relaxation Enhancement. J. Am. Chem. Soc. 132 (16), 5546–5547. 10.1021/ja9105495 20359221 PMC3064441

[B159] Xu M.-M. Ryan P. Rudrawar S. Quinn R. J. Zhang H.-Y. Mellick G. D. (2020). Advances in the Development of Imaging Probes and Aggregation Inhibitors for Alpha-Synuclein. Acta Pharmacol. Sin 41 (4), 483–498. 10.1038/s41401-019-0304-y 31586134 PMC7470848

[B160] Yu H. Han W. Ma W. Schulten K. (2015). Transient β-hairpin Formation in α-synuclein Monomer Revealed by Coarse-Grained Molecular Dynamics Simulation. J. Chem. Phys. 143 (24), 243142. 10.1063/1.4936910 26723627 PMC4684271

[B161] Zarranz J. J. Alegre J. Gómez-Esteban J. C. Lezcano E. Ros R. Ampuero I. (2004). The New Mutation, E46K, of α-synuclein Causes Parkinson and Lewy Body Dementia. Ann. Neurol. 55 (2), 164–173. 10.1002/ana.10795 14755719

[B162] Zhang Y. Hashemi M. Lv Z. Williams B. Popov K. I. Dokholyan N. V. (2018). High-speed Atomic Force Microscopy Reveals Structural Dynamics of α-synuclein Monomers and Dimers. J. Chem. Phys. 148 (12), 123322. 10.1063/1.5008874 29604892 PMC5764752

[B163] Zigoneanu I. G. Yang Y. J. Krois A. S. Haque M. E. Pielak G. J. (2012). Interaction of α-synuclein with Vesicles that Mimic Mitochondrial Membranes. Biochim. Biophys. Acta (Bba) - Biomembr. 1818 (3), 512–519. 10.1016/j.bbamem.2011.11.024 PMC3273638 22155643

